# Maternal Excessive Lard Versus Palm Oil Intake in Mice Drives Sex-Specific Reproductive Dysfunction in Offspring

**DOI:** 10.3390/nu18162631

**Published:** 2026-08-12

**Authors:** Yan Xu, Yue Zhang, Kaile Guan, Qi Chen, Chenchen Geng, Lingfeng Dan, Jiaxin Zhao, Yansong Zhang, Huimin Lu

**Affiliations:** 1 Department of Nutrition and Food Hygiene (National Key Discipline), School of Public Health, Harbin Medical University, Harbin 150081, China; 202301102@hrbmu.edu.cn (Y.X.); 2024020259@hrbmu.edu.cn (Y.Z.); 2023020164@hrbmu.edu.cn (K.G.); 2024020165@hrbmu.edu.cn (Q.C.); 2022020171@hrbmu.edu.cn (C.G.); 2021020221@hrbmu.edu.cn (L.D.); 2025020133@hrbmu.edu.cn (J.Z.); 2025020209@hrbmu.edu.cn (Y.Z.); 2 Key Laboratory of Precision Nutrition and Health of Ministry of Education, School of Public Health, Harbin Medical University, Harbin 150081, China

**Keywords:** maternal high-saturated-fat diets, offspring, reproductive health, sex-specific difference, transcriptomic sequencing, saturated fat source

## Abstract

**Objectives**: This study clarified sex-specific short- and long-term impacts of maternal excessive palm oil (plant-derived saturated) and lard (animal-derived saturated fat) intake on offspring reproductive health and underlying mechanisms. **Methods**: Female mice were randomized to control, palm oil, or lard diets during gestation and lactation. Offspring received a control diet post-weaning until adulthood, followed by an optional 16-week HFD rechallenge. Gonadal weight and organ index, sperm quality, ovarian follicle number, serum sex hormones, metabolic phenotypes (serum lipid profiles, glucose and insulin tolerance), oxidative stress, apoptosis, proliferation, and transcriptomic profiles were detected at weaning, early and late adulthood. **Results**: In males, excessive maternal lard intake significantly induces sperm malformation and reduces seminiferous tubule diameter and spermatogenic epithelium thickness upon HFD rechallenge during adulthood, with no changes in oxidative stress, proliferation, and apoptosis. Transcriptomics identified *Slc24a5* upregulation as a potential correlate through metal ion transmembrane transporter activity and cellular calcium ion homeostasis. Under normal adult diets, reduced sperm motility resulting from maternal excess lard may be mediated by apoptosis, whereas that induced by maternal high palm oil likely arises from suppressed proliferation and thinner spermatogenic epithelium. GO analysis revealed palm oil-specific *Ugt1a5* upregulation associated with altered steroid hormone metabolism. In females, maternal palm oil intake reduced primordial follicles and increased atresia, with sequencing predicted to involve *Nedd4* downregulation; lard intake elevated ovarian oxidative stress and atresia only upon HFD rechallenge, with *Lamc3* upregulation predicted as a putative regulatory factor. **Conclusions**: Maternal excessive intake of saturated fat exerts offspring reproductive damage in a sex-specific and source-dependent manner with distinct mechanisms. This work provides novel insights into fat source-dependent and sex-specific effects of nutritional interventions during pregnancy and lactation.

## 1. Introduction

Global decline in fertility has raised widespread concerns regarding reproductive health, including reduced male sperm quality, polycystic ovary syndrome, and premature ovarian insufficiency [1,2,3]. As public awareness of reproductive health continues to grow, increasing research efforts have focused on identifying fertility-related risk factors and developing effective preventive strategies. Accumulating evidence indicates that environmental exposure and unhealthy lifestyles adversely affect reproductive health [4]. Importantly, adverse exposures during early life, particularly during maternal pregnancy and lactation, can induce irreversible reproductive damage in offspring [5].

A balanced maternal diet is critical for fetal development, and nutritional imbalance during the intrauterine and early postnatal periods increases the risk of subfertility in offspring [6]. High-fat diets (HFD) represent a prominent risk factor for embryonic and neonatal health [7]. Many women tend to consume excessive high-fat foods during pregnancy and lactation under the misconception that this practice benefits nutritional supplementation. However, emerging evidence confirms that maternal HFD exposure triggers metabolic disorders and nutritional stress, thereby leading to offspring obesity, gonadal damage, and elevated infertility risk in adulthood [8,9].

Excessive intake of saturated fat is a key driver of obesity and related diseases [10]. Beyond total saturated fat quantity, emerging evidence highlights that the saturated-fat source (quality) exerts divergent effects on disease pathogenesis. Animal-derived saturated fat (e.g., lard) and plant-derived (e.g., palm oil, coconut oil, cocoa powder) substantially differ in full fatty acid profiles, minor bioactive constituents, and downstream metabolic actions; accordingly, their overconsumption may elicit distinct health outcomes [11,12,13]. Despite this heterogeneity, the vast majority of preclinical HFD models adopt lard as the primary fat source, largely overlooking plant-based saturated fat alternatives.

This study clarifies how maternal saturated fat intake (by source) affects offspring reproductive health, comparing short- (early childhood) and long-term (adulthood) effects and highlighting sex-specific and stage-specific differences. It elucidates the role of maternal saturated fat quality in offspring reproductive development and provides theoretical support for optimizing maternal dietary strategies.

## 2. Materials and Methods

### 2.1. Animals and Experimental Design

**Figure 1:**
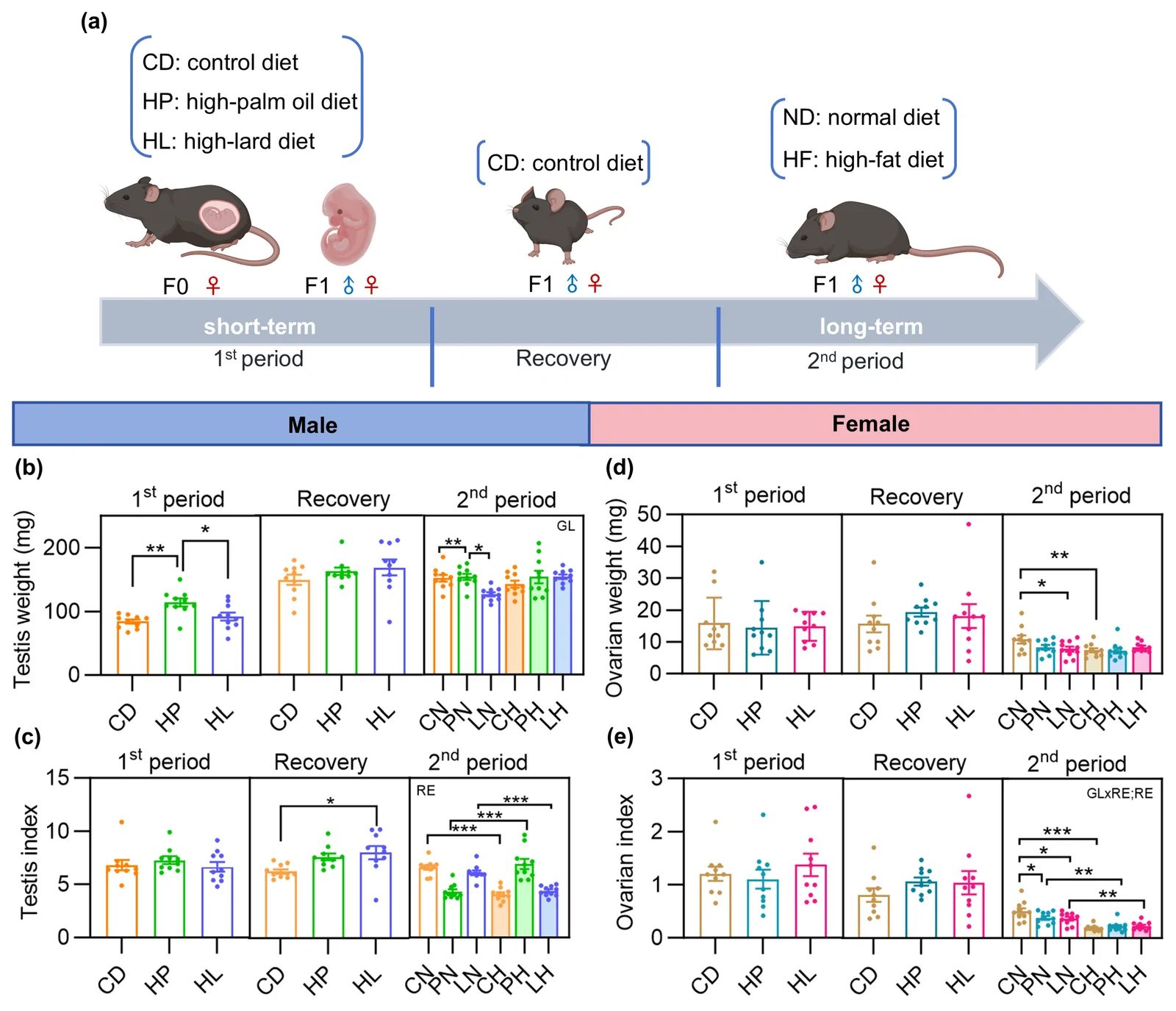
Effects of maternal excessive palm oil or lard intake during gestation and lactation on offspring gonadal weight and index (sex-specific, short- and long-term): (**a**) Workflow of the animal experiments: Dams were fed CD, HP, HL diets during gestation and lactation; At weaning, the offsprings were given CD until early adult (12-week-old); In late adulthood, the offspring were fed ND or HF for 16 weeks. (**b**) Absolute testicular weight; (**c**) Testicular index; (**d**) Absolute ovarian weight; (**e**) Ovarian index. 1st period: Maternal exposure to HFD during gestation and lactation; Recovery: offspring were fed a control diet until early adulthood; 2nd period: Re-exposure to HFD in late adulthood. * *p* < 0.05, ** *p* < 0.01, *** *p* < 0.001 by one-way ANOVA at 1st period and Recovery and two-way ANOVA at 2nd period. For 1st period, Recovery, and 2nd period: *n* = 10 mice per group. GL: effect of HFD in gestation and lactation; RE: effect of HFD re-exposure; GL × RE: interactive effect of GL and RE. For 1st period and Recovery: CD, control diet; HP, high-palm-oil diet; HL, high-lard diet. For 2nd period: CN, PN, LN, first exposure to CD/HP/HL and re-exposure to normal diet; CH, PH, LH, first exposure to CD/HP/HL and re-exposure to high-fat diet.

C57BL/6N mice (female, 6–8 weeks old, body weight: 16.46 ± 1.07 g, Beijing Vital River, Beijing, China) were housed under standard conditions (12/12 h light/dark cycle, 22 ± 2 °C, ad libitum food and water). After 1 week of acclimatization, female mice were mated with stud males at a 2:1 ratio. To minimize batch effects, only mice with vaginal plugs detected within the first three days after mating were included. The presence of a vaginal plug was designated as gestational day 0 (GD0). Pregnant females were then randomly assigned to three experimental diets throughout gestation and lactation: control (15.75% energy from soybean oil, SY93G), high palm oil (60% energy from palm oil, SYHPA60), and high lard (60% energy from lard, SYHSA60). After parturition, litter size was standardized to 6 pups per litter (3 males, 3 females) on postnatal day 4 (PND4) to ensure consistent lactation nutrition. Dams that could not meet the standard litter gender ratio were further excluded. Pups that died naturally, presented physical malformations, growth retardation, or extreme body weight deviation were excluded. All surplus unselected pups were humanely euthanized by trained technicians via cervical dislocation, a procedure that fully severs the spinal cord at the atlanto-occipital joint, in accordance with the 2020 AVMA guidelines. All animals were monitored for a minimum of 60 s to confirm irreversible death via sustained apnea and loss of the corneal reflex prior to tissue harvesting. At weaning (PND28), offspring from each maternal group were allocated into three cohorts for tissue collection and measurements at three separate time points: weaning, early adulthood (after 8 weeks on control diet), and late adulthood (following 16 weeks of normal diet ‘H10010’ or high-fat diet challenge, 45% kcal from fat, H10045) (Figure 1a). All male and female pups underwent the same experimental manipulations, with sex-stratified statistical analyses conducted to evaluate sex-specific phenotypic disparities. To minimize litter bias, no more than one male and one female pup from each litter were assigned to the same experimental group. Litter was defined as the experimental unit for statistical analysis, and only one mouse was randomly selected from each litter for detection, so each mouse represented an independent litter without pseudoreplication. Sample size was calculated a priori using G*Power 3.1 according to a two-way ANOVA design (α = 0.05, power = 0.80, effect size f = 0.25) [14,15]. At each time point, 12 offspring mice were initially assigned per group at study onset, according to the 3R principles and practical animal availability. Body weight and gonadal weights (testes/ovaries) were measured, and serum, gonads, and sperm samples were collected at each time point. This study was approved by the Institutional Animal Care and Use Committee of Harbin Medical University (HMUIRB2022024 and HMUIRB2026022PRE) and conducted per institutional guidelines. The diets during gestation and lactation were purchased from Shuyu Biotech, Shanghai, China. The diets for re-exposure were from Beijing HFK BIOSCIENCE Co., Ltd., Beijing, China. The ingredients and fatty acid profiles of all diets were listed in Supplementary Tables S1, S2, S3, and S4, respectively.

### 2.2. Determination of Sperm Concentration, Motility and Malformation Rate

The right epididymis tail of adult mice was minced in 37 °C saline, incubated for 15 min, and gently shaken to prepare sperm suspension. Sperm concentration was counted using a hemocytometer, and motility was evaluated via video recording (mean of 4 independent fields per sample). For malformation rate, 10 μL suspension was smeared, HE-stained, and ≥200 sperm/sample were observed under a light microscope to record abnormal sperm number and rate.

### 2.3. HE Staining and Morphometric Analysis of Ovaries and Testes

Ovaries and testes were fixed in 4% paraformaldehyde, dehydrated in graded ethanol, paraffin-embedded, and sectioned (5 μm). Sections were HE-stained, imaged with a digital scanning microscope (M8), and analyzed via ImageJ 1.54P. For testes: 50 intact, clear-lumen circular/near-circular seminiferous tubule cross-sections were selected per sample; tubule diameter (shortest basement membrane–lumen distance) and epithelial thickness (mean vertical distance at 4 equidistant points) were measured. For ovaries: follicles were classified into 5 types (primordial, primary, secondary, atretic, corpora lutea). Five representative sections per ovary were used to count nucleated oocyte-containing follicles (to avoid duplication). Quantifications included type-specific and total follicle counts, corpora lutea per mouse, and atresia rate (atretic/total follicles ratio).

### 2.4. RNA Sequencing and Data Analysis

Testes and ovaries (3–5 samples/group at each time point; sample size was determined based on previous studies [16,17]) were collected at weaning, 8-week recovery, and 16-week HFD rechallenge for RNA sequencing (Shanghai Biotree Biotech, Shanghai, China). Two ovaries collected from a single mouse (the left and right ovary of the same animal) were pooled to obtain sufficient RNA yield. RNA integrity was evaluated using an Agilent 2100 Bioanalyzer (Agilent Technologies, Santa Clara, CA, USA). Samples with RNA integrity number (RIN) > 7.0, concentration > 50 ng/μL, and total RNA > 1 μg were subjected to library preparation. Libraries were constructed with Illumina Truseq^TM^ RNA Kit (Illumina Inc., San Diego, CA, USA) and sequenced on the Illumina NovaSeq platform. Raw sequencing reads were quality-checked with FastQC (v0.10.1) and trimmed for adapters and low-quality sequences using cutadapt (v.1.9). Clean reads were aligned to the mouse reference genome (Ensembl v112; GRCm39/mm39) via HISAT2 (v2.2.1). Transcript assembly and gene quantification were conducted using StringTie (v2.1.6), and differential expression analysis was performed with DESeq2 (v1.22.2) based on pairwise group comparisons. Genes with |log_2_fold change (FC)| ≥ 1 and adjusted *p*-value (q) < 0.05 were defined as differentially expressed. We further performed Gene Ontology (GO) and Kyoto Encyclopedia of Genes and Genomes (KEGG) pathway enrichment analyses, as well as Search Tool for the Retrieval of Interacting Genes/Proteins (STRING)-based protein-protein interaction (PPI) network analysis.

### 2.5. Determination of Serum Sex Hormone Levels

Serum testosterone (TT), follicle-stimulating hormone (FSH), and estradiol (E_2_) levels were measured using ELISA kits (testosterone, MM-0569M1; FSH, MM-45654M1; estradiol, MM-0566M1; Jiangsu Meimian Industrial Co., Ltd., Yancheng, Jiangsu, China). The assay was performed strictly in accordance with the manufacturer’s instructions. The limits of detection (LOD) for TT, FSH, and E_2_ were 0.0375 nmol/mL, 0.125 U/L, and 2 pmol/mL, respectively.

### 2.6. Determination of Antioxidant Enzyme Activity

Mouse testes or ovaries were thoroughly rinsed with cold saline and homogenized in saline at a 1:9 weight-to-volume ratio (g:mL) on ice. Following low-temperature centrifugation, the supernatant was collected for subsequent analysis. Superoxide dismutase (SOD) activity was measured using the water-soluble tetrazolium salt (WST-1) method [18,19]. Assays were performed with a commercial kit (Cat. No. A001-3-2, Nanjing Jiancheng Bioengineering Institute, Nanjing, China), and all procedures strictly followed the manufacturer’s instructions. Glutathione peroxidase (GPX) activity was determined using the 5,5′-dithiobis-(2-nitrobenzoic acid) (DTNB) method [20,21]. A commercial kit (Cat. No. A005-1-2, Nanjing Jiancheng Bioengineering Institute, Nanjing, China) was used according to the manufacturer’s protocol.

### 2.7. TUNEL Staining for Apoptosis Detection

Testicular apoptosis was detected by chromogenic TUNEL assay (WAS1905, WASci Biotechnology, Shanghai, China). Paraffin sections were deparaffinized, rehydrated, permeabilized with proteinase K (20 μg/mL), incubated with TdT-biotin-dUTP, then streptavidin-HRP and DAB chromogen. Sections were counterstained with hematoxylin, differentiated, and mounted. Tubules with ≥3 brown apoptotic nuclei were counted; apoptosis rate = positive tubules/total tubules. Ovarian apoptosis was analyzed via direct fluorescent TUNEL assay (11684795910, Roche, Basel, Switzerland). After deparaffinization, rehydration, and permeabilization, sections were incubated with TUNEL reaction mixture (enzyme:labeling solution = 1:9), washed, counterstained with DAPI, and mounted with anti-quenching medium. Four to five non-overlapping ×400 fields/section were imaged; TUNEL-positive (green) and total (blue) nuclei were counted by ImageJ to calculate the apoptosis rate.

### 2.8. Cell Proliferation Assay

Proliferation in testes or ovaries was detected by Ki67 immunohistochemical staining. Paraffin sections were dewaxed, hydrated, treated with H_2_O_2_ blocking solution, then antigen-retrieved in boiling Tris-EDTA and cooled naturally. Sections were blocked with 5% BSA, incubated with Ki67 primary antibody (ab16667, Abcam, Cambridge, UK), followed by HRP-conjugated secondary antibody (WAS12011, 1:200, 37 °C, 30 min) and DAB development (terminated upon signal appearance). Sections were counterstained with hematoxylin, differentiated (acid alcohol), blued, dehydrated, cleared, and mounted. Images were analyzed by ImageJ; proliferation was presented as the percentage of Ki67-positive area.

### 2.9. qPCR

Total RNA was extracted from mouse testes and ovaries using the M5 Universal RNA Mini Kit (Catalog No. MF036-01, Mei5 Biotechnology, Beijing, China). RNA was reverse-transcribed with the M-MLV RT RNase (H^−^) Point Mutant (Catalog No. M368C, Promega, Madison, WI, USA), and quantitative amplification was performed using the PowerUp SYBR Green Master Mix (Catalog No. A25742, Applied Biosystems, Waltham, MA, USA). Primers were synthesized by Sangon Biotech (Shanghai, China), and all primer sequences are provided in Supplementary Table S5.

### 2.10. Statistical Analysis

Data were analyzed with SPSS 26.0 and expressed as mean ± SEM. A *p*-value < 0.05 was considered statistically significant. One-way ANOVA was used for analyses at the weaning or recovery stages. Two-way ANOVA assessed the interaction between maternal and offspring dietary patterns. Bonferroni’s post hoc test was performed for pairwise inter-group comparisons to control the Type I error rate caused by multiple repeated comparisons of each individual biological indicator.

## 3. Results

### 3.1. Maternal Excessive Palm Oil or Lard Intake During Pregnancy and Lactation Significantly Reduces Female (Not Male) Offspring Ovarian Index

At weaning (1st period), male offspring testicular weight was higher in the maternal high-palm oil than in control and high-lard groups. This difference disappeared after 8 weeks on the post-weaning control diet (Recovery) but re-emerged at 16 weeks with lower testicular weight versus control and high-lard groups (2nd period) (Figure 1b). Testicular index changes were inconsistent: maternal high-saturated-fat diets had no effect at weaning, but decreased significantly upon adult HFD re-exposure, independent of maternal diet (Figure 1c). In contrast, maternal high-saturated-fat diet did not affect ovarian weight or index of female offspring at weaning or 8 weeks of control diet post-weaning, but both decreased significantly in late adulthood under normal diet. This late-adult decrease showed no difference by maternal diet (Figure 1d,e). These results suggest that maternal excessive palm oil or lard intake exerts more pronounced, long-term effects on female offspring gonads (reduced weight and index), with no further aggravation following HFD re-exposure.

### 3.2. Trajectory of Maternal Excess Palm Oil or Lard Intake Effects on Offspring Sperm and Gonadal Structures

**Figure 2:**
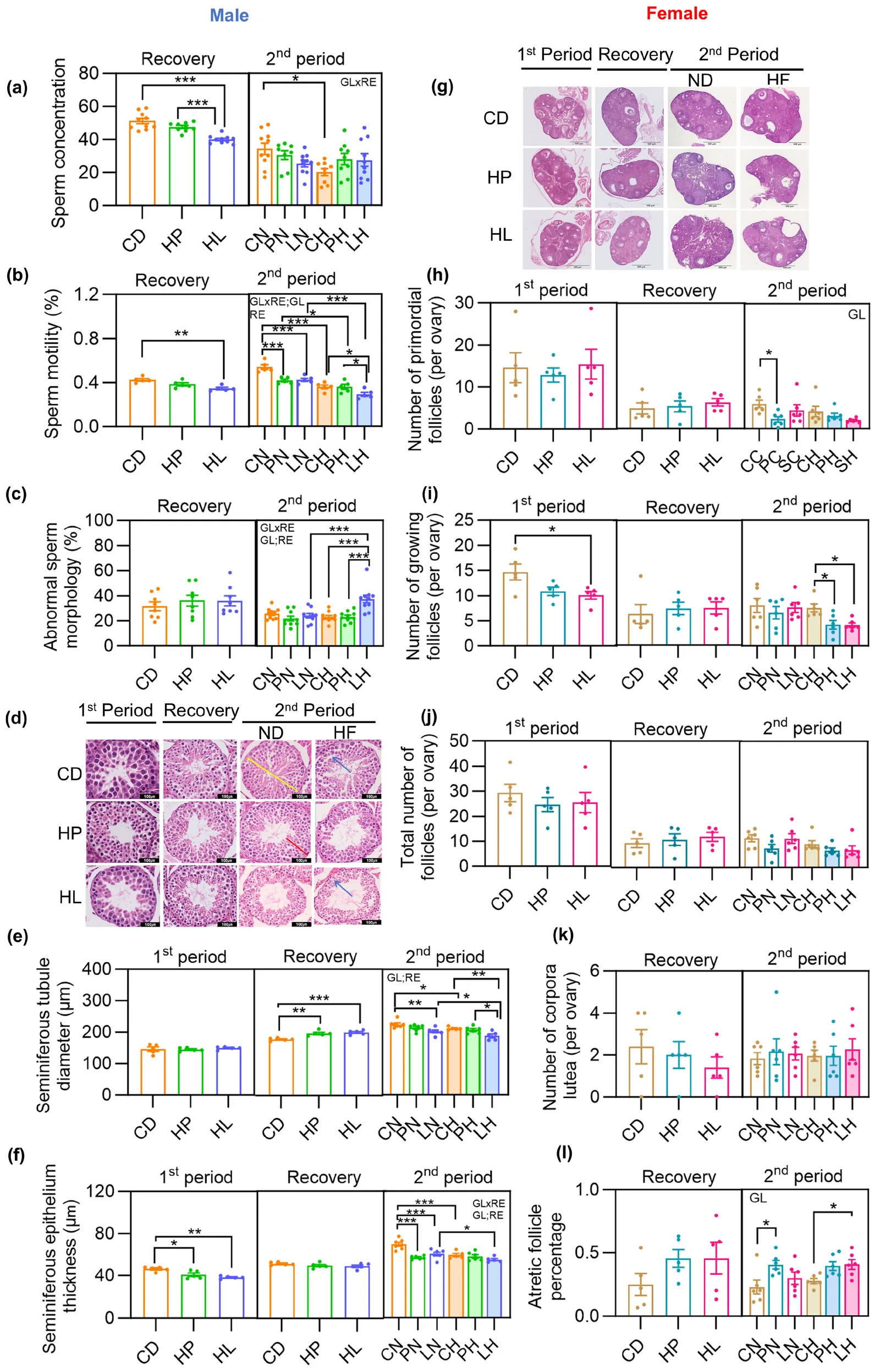
Effects of maternal excessive palm oil or lard intake during pregnancy and lactation on offspring sperm parameters (motility, concentration) and gonadal histomorphology: (**a**) Sperm concentration; (**b**) Sperm motility; (**c**) Percentage of abnormal sperm morphology; (**d**) Hematoxylin-eosin (HE) staining of testes; the yellow line indicates seminiferous tubule diameter, the red line indicates spermatogenic epithelium thickness, and blue arrows indicate partial loss of spermatogenic cells; (**e**) Seminiferous tubule diameter; (**f**) Seminiferous epithelium thickness; (**g**) Hematoxylin-eosin (HE) staining of ovaries; (**h**) Number of primordial follicles; (**i**) Number of growing follicles; (**j**) Total number of follicles; (**k**) Number of corpora lutea; (**l**) Atretic follicle percentage. 1st period: Maternal exposure to HFD during gestation and lactation; Recovery: offspring were fed a control diet until early adulthood; 2nd period: Re-exposure to HFD in late adulthood. * *p* < 0.05, ** *p* < 0.01, *** *p* < 0.001. One-way ANOVA was used for the 1st period and Recovery. Two-way ANOVA was used for the 2nd period. For (**a**), Recovery: CD, *n* = 10; HP, *n* = 9; HL, *n* = 10; 2nd period: CN, *n* = 10; PN, *n* = 8; LN, *n* = 10; CH, *n* = 9; PH, *n* = 9; LH, *n* = 10. For (**b**), *n* = 5 mice per group. For (**c**), Recovery: *n* = 8 per group; 2nd period: *n* = 10 per group. For (**e**,**f**), 1st period: *n* = 5 per group; Recovery: *n* = 5 per group; 2nd period: CN, *n* = 7; PN, *n* = 6; LN, *n* = 6; CH, *n* = 5; PH, *n* = 6; LH, *n* = 5. For (**h**–**j**), 1st period: CD, *n* = 5 per group; Recovery: CD, *n* = 5; HP, *n* = 5; HL, *n* = 6; 2nd period: *n* = 6 per group. For (**k**,**l**), Recovery: CD, *n* = 5; HP, *n* = 5; HL, *n* = 6; 2nd period: *n* = 6 per group. GL: effect of HFD in gestation and lactation; RE: effect of HFD re-exposure; GL × RE: interactive effect of GL and RE. For 1st period and Recovery: CD, control diet; HP, high-palm oil diet; HL, high-lard diet. For 2nd period: CN, PN, LN, first exposure to CD/HP/HL and re-exposure to normal diet; CH, PH, LH, first exposure to CD/HP/HL and re-exposure to high-fat diet; ND, normal diet; HF, high-fat diet.

Maternal excessive lard intake during gestation and lactation reduced sperm concentration (Figure 2a) and motility (Figure 2b) after recovery, which was not observed in the high-palm-oil group. With aging, sperm motility decreased in maternal high-saturated-fat-exposed males after switching to a control diet (Figure 2b). HFD re-exposure revealed that maternal excessive lard intake increased sperm abnormality and inhibited motility (Figure 2b,c). HE staining showed that maternal high-saturated-fat diets induced loose testicular tissue, disordered spermatogenic cells, germ cell loss, and reduced spermatogenic epithelium thickness (no change in seminiferous tubule diameter). A normal diet restored spermatogenic epithelium thickness but increased seminiferous tubule diameter. HFD re-exposure exacerbated lard-induced tubule diameter reduction and epithelium thinning (Figure 2d,f). In females, maternal excessive palm oil intake reduced the number of primordial follicles and increased atretic follicles under a normal diet. HFD re-exposure increased atretic follicles in the maternal high-lard group but not the high-palm oil group (Figure 2g–l). These results indicate maternal high-saturated-fat diets impair gonadal structure in offspring of both sexes: males are more sensitive to maternal excessive lard intake (sperm and testicular damage), while females exhibit impaired follicle number with both high-palm oil and high-lard intake.

### 3.3. Maternal Excess Palm Oil or Lard Intake on Offspring Serum Hormones, Glucose and Insulin Tolerance, Fatty Acid Profile, and Lipid Levels

**Figure 3:**
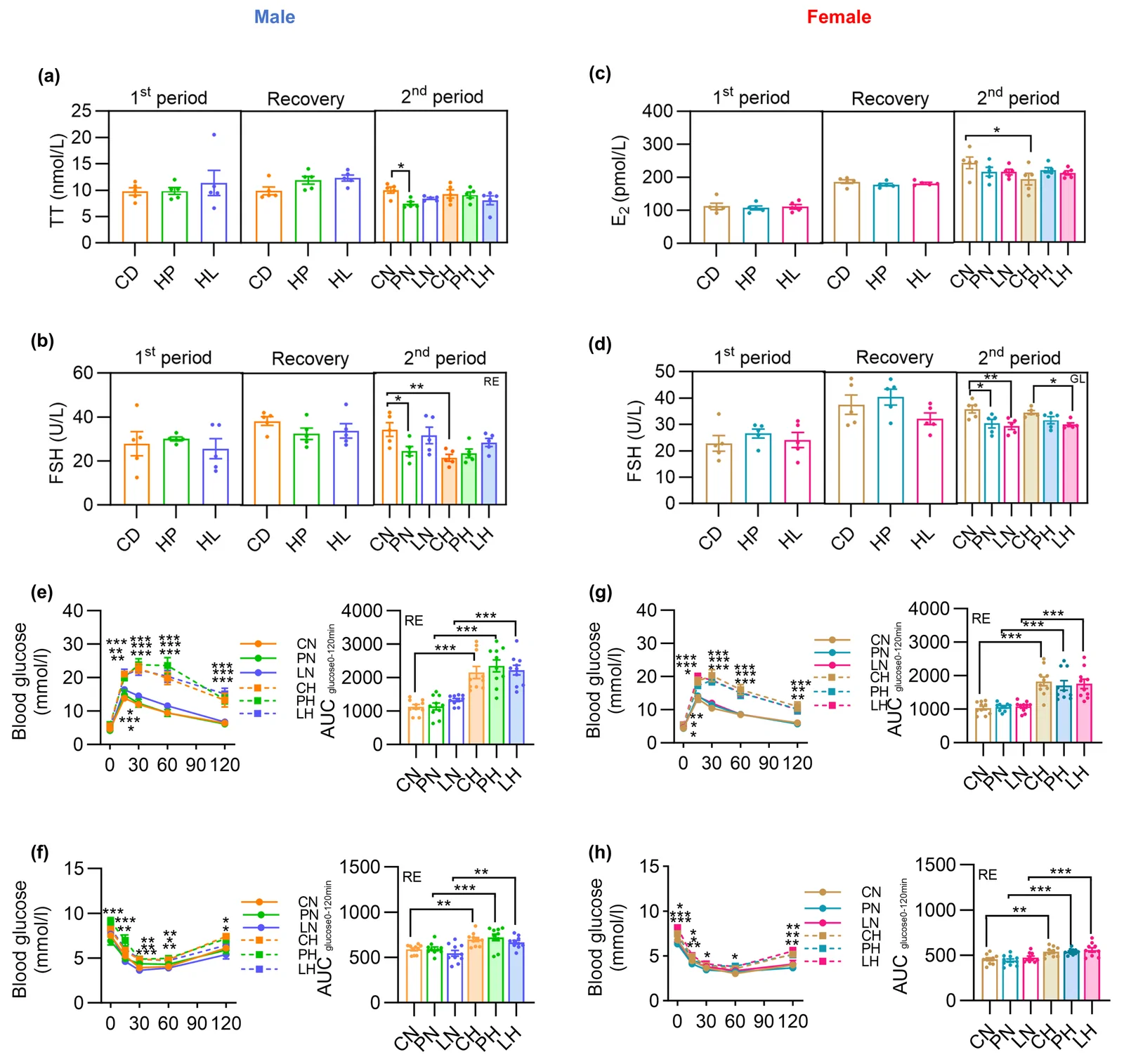
Effects of maternal excessive palm oil or lard intake during pregnancy and lactation on offspring serum hormones and glucose/insulin tolerance: (**a**,**b**) Serum TT and FSH levels in male offspring; (**c**,**d**) E2 and FSH levels in female offspring. EBlood. Experimental periods: 1st period: maternal exposure to high-fat diet during gestation and lactation; Recovery, offspring were fed a control diet until early adulthood; 2nd period: re-exposure to HFD in late adulthood. * *p* < 0.05, ** *p* < 0.01. One-way ANOVA was used for the 1st period and Recovery. Two-way ANOVA was used for the 2nd period. For (**a**–**d**), *n* = 5 mice per group. For (**e**–**h**), *n* = 10 mice per group. For the curve in (**e**–**h**), * *p* < 0.05, ** *p* < 0.01, *** *p* < 0.001, compared to corresponding controls. GL: effect of HFD exposure during gestation and lactation; RE: effect of HFD re-exposure. For 1st period and Recovery: CD, control diet; HP, high-palm-oil diet; HL, high-lard diet. For 2nd period: CN, PN, LN, first exposure to CD/HP/HL and re-exposure to normal diet; CH, PH, LH, first exposure to CD/HP/HL and re-exposure to high-fat diet.

In males, maternal high-palm-oil exposure reduced circulating TT and FSH levels, and this effect was not modified by HFD re-exposure (Figure 3a,b). In females, E_2_ concentrations were unaffected by maternal diets. By contrast, FSH was decreased in both maternal high-palm-oil and -lard groups; upon HFD re-exposure, FSH was significantly lower than controls exclusively in the maternal high-lard group (Figure 3c,d). Additionally, impairments in glucose and insulin tolerance triggered by HFD re-exposure were independent of maternal dietary conditions (Figure 3e–h). Serum fatty acid profiling revealed that among male offspring (Supplementary Table S6), the concentrations of palmitic acid, palmitoleic acid, stearic acid, oleic acid, and total saturated fatty acids, together with the proportion of stearic acid and the stearic-to-palmitic acid ratio, were selectively elevated in the LH group. In female offspring (Supplementary Table S7), no significant differences were detected between offspring exposed to maternal high-palm-oil and lard diets. Analysis of serum lipid parameters indicated no disparities between PN versus LN and PH versus LH in male offspring (Supplementary Table S8). By contrast, female offspring in the PH group exhibited markedly lower serum lipid levels (Supplementary Table S9). Collectively, these metabolic findings suggest that distinct reproductive dysfunction triggered by maternal overconsumption of saturated fats from different sources arises from disrupted serum fatty acid metabolism, rather than altered glucose and lipid homeostasis.

### 3.4. Stage-Specific Trajectory of Maternal Excess Palm Oil or Lard Intake on Gonadal Antioxidant Activity, Apoptosis and Proliferation

**Figure 4:**
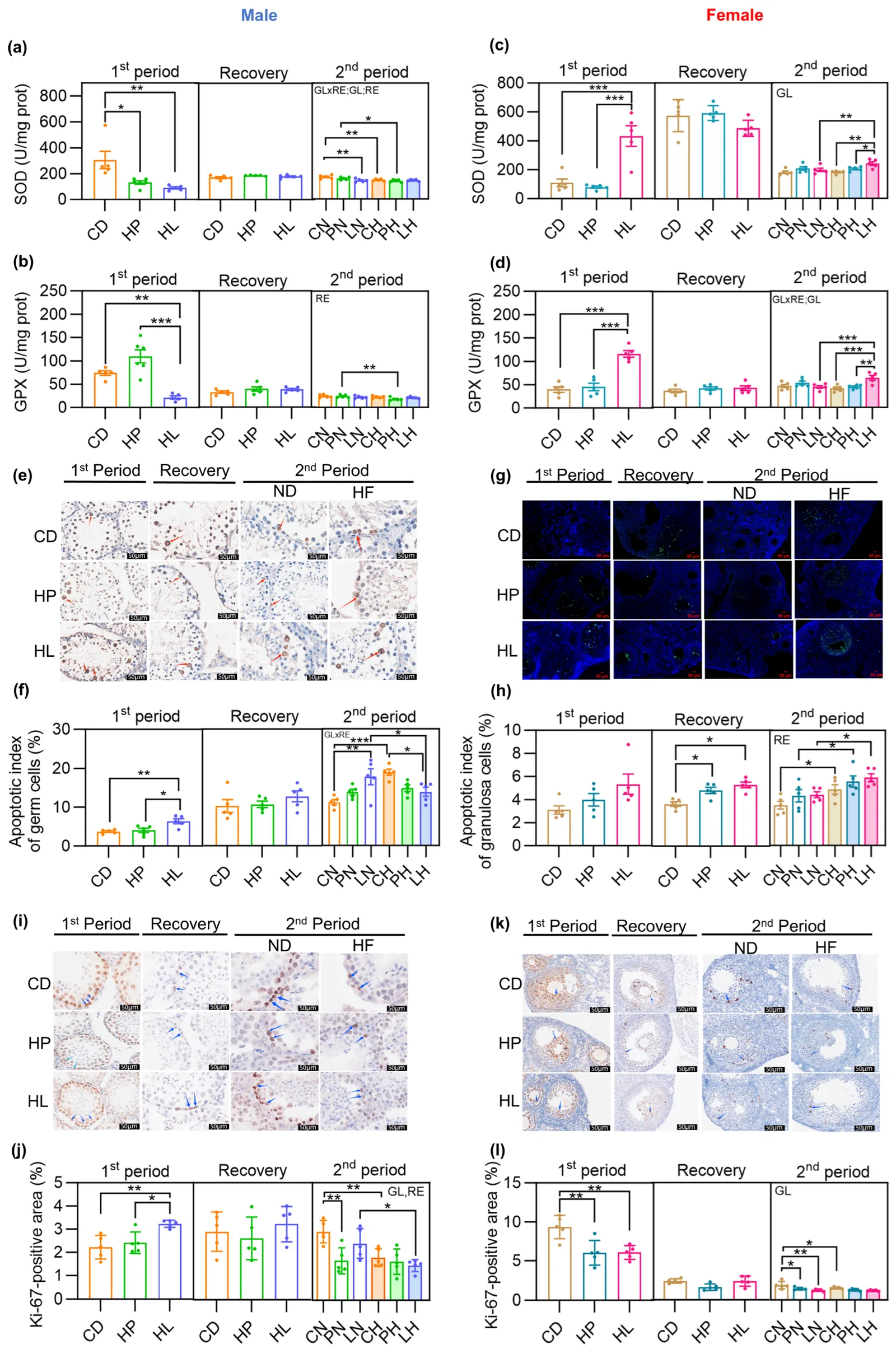
Effects of maternal excessive palm oil or lard intake during pregnancy and lactation on offspring gonadal antioxidant activity, apoptosis and proliferation: (**a**,**b**) SOD and GPX levels in testes; (**c**,**d**) SOD and GPX levels in ovaries; (**e**) Light micrographs of testicular sections showing TUNEL-positive apoptotic germ cells (arrows); (**f**) Apoptotic index of germ cells (%) in the testes; (**g**) Fluorescence micrographs of ovarian sections showing apoptotic germ cells (arrows); (**h**) Apoptotic index of granulosa cells (%) in the ovaries; (**i**) Ki-67 immunohistochemical staining of testicular sections; arrows indicate Ki-67-positive cells; (**j**) Percentage of Ki-67-positive area in testicular tissues; (**k**) Ki-67 immunohistochemical staining of ovarian sections; arrows indicate Ki-67-positive cells; (**l**) Percentage of Ki-67-positive area in ovarian tissues. All scale bars indicate 50 μm. Experimental periods: 1st period: maternal exposure to a high-fat diet during gestation and lactation; Recovery: offspring were fed a control diet until early adulthood; 2nd period: re-exposure to HFD in late adulthood. * *p* < 0.05, ** *p* < 0.01, *** *p* < 0.001. One-way ANOVA was used for the 1st period and Recovery. Two-way ANOVA was used for the 2nd period. For (**a**,**b**), 1st period: CD, *n* = 5; HP, *n* = 6; HL, *n* = 5. For Recovery: *n* = 5 mice per group. For 2nd period: *n* = 5 mice per group. For (**c**,**d**,**f**,**h**,**j**,**l**), *n* = 5 mice per group. GL: effect of HFD exposure during gestation and lactation; RE: effect of HFD re-exposure; GL × RE: interactive effect of GL and RE. For 1st period and Recovery: CD, control diet; HP, high-palm-oil diet; HL, high-lard diet. For 2nd period: CN, PN, LN, first exposure to CD/HP/HL and re-exposure to normal diet; CH, PH, LH, first exposure to CD/HP/HL and re-exposure to high-fat diet; ND, normal diet; HF, high-fat diet.

Maternal excess lard intake altered infant male testicular and female ovarian SOD/GPX levels (decreased in testes, increased in ovaries), and this difference disappeared after 8 weeks of control diet. In adult males (additional 16 weeks of normal diet), testicular SOD decreased again; in females, ovarian SOD/GPX increased again upon HFD re-exposure (Figure 4a–d). Maternal excess palm oil intake only reduced male testicular SOD at weaning (Figure 4a). Apoptosis assays showed maternal excess lard intake increased weaning male testicular apoptotic rates. Post-control diet recovery, testicular apoptosis recovered, but ovarian apoptosis emerged in both lard and palm oil groups. Maternal high-saturated-fat intake did not exacerbate adult HFD re-exposure-induced apoptosis (Figure 4e–h).

Cell proliferation assays showed maternal excess lard intake enhanced cell proliferation in infant male testes; this effect disappeared post-control diet. By contrast, maternal high palm oil intake inhibited testicular proliferation in adult male offspring maintained on a 16-week normal diet. In females, both maternal excess lard and palm oil intake inhibited ovarian proliferation at weaning. This inhibitory effect disappeared following 8-week control-diet feeding but re-emerged in late adulthood (Figure 4i–l).

### 3.5. Transcriptomic Regulatory Networks in Offspring Gonads Following Maternal Excess Palm Oil or Lard Intake

**Figure 5:**
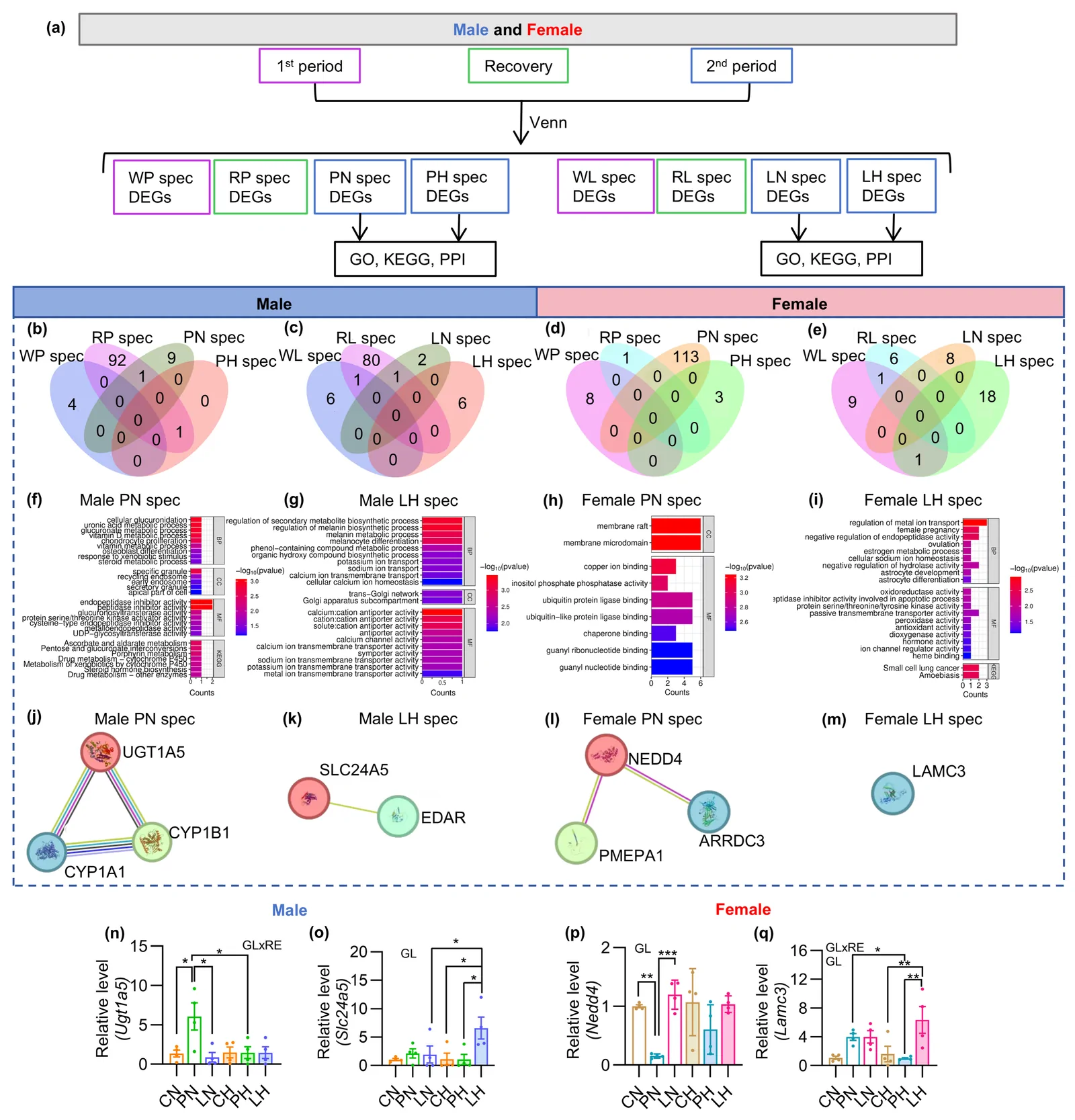
Effects of maternal excess palm oil or lard intake during pregnancy and lactation on transcriptomes of testes and ovaries in offspring mice: (**a**) Flow chart of differential gene screening; (**b**–**e**) Venn analysis of testes and ovaries across different stages; (**f**–**i**) GO, KEGG analyses of DEGs in the PN and LH spec groups in both male and female offspring; (**j**–**m**) PPI analyses in offsprings of both sexes. (**n**–**q**) Expression of *Ugt1a5*, *Slc24a5* in testes and *Nedd4*, *Lamc3* in ovaries. Experimental periods: 1st period: maternal exposure to high-fat diet (HFD) during gestation and lactation; Recovery, offspring were fed a control diet until early adulthood; 2nd period: re-exposure to HFD in late adulthood. WP or WL spec, DEGs specific to maternal high palm oil or high lard at weaning; RP or RL spec, DEGs specific to maternal high palm oil or high lard after recovery of an 8-week control diet; PN or LN spec, DEGs specific to maternal high palm oil or lard after second period of normal diet; PH or LH spec, DEGs specific to maternal high-palm oil or high-lard after second period of HFD. For testes RNA-Seq (**b**,**c**), at weaning, *n* = 5 in WP and *n* = 4 in WL; *n* = 5 in RP and RL; *n* = 3 in PN/PH/LN and *n* = 4 in LH. For ovary RNA-Seq (**d**,**e**), at weaning, *n* = 5 in WP and *n* = 4 in WL; *n* = 5 in RP and RL; *n* = 3 in PN/PH/LN and *n* = 4 in LH. For qPCR (**n**–**q**), *n* = 4 mice per group. * *p* < 0.05, ** *p* < 0.01, *** *p* < 0.001 by two-way ANOVA of. GL: effect of HFD exposure during gestation and lactation; RE: effect of HFD re-exposure; GL × RE: interactive effect of GL and RE. CN, PN, LN, first exposure to CD/HP/HL and re-exposure to normal diet; CH, PH, LH, first exposure to CD/HP/HL and re-exposure to high-fat diet.

To clarify sexual dimorphism in gonadal damage sensitivity, transcriptomic sequencing was performed on offspring testes (males) and ovaries (females) at three life stages: weaning, early adulthood (post-control diet), and late adulthood (HFD re-exposure). Figure 5a shows the bioinformatic workflow and differentially expressed genes (DEGs) screening. Stage-specific DEGs induced by maternal high-palm oil or high-lard diets were identified (Supplementary Figure S1), and Venn analysis of the same diet DEGs across stages (Figure 5b–e) yielded DEGs matching sex-specific phenotypes.

In males, maternal high-palm oil diet altered 4 testicular DEGs at weaning, 94 at early adulthood, 10 at late adulthood (PN-specific), and 1 at HFD re-exposure (PH-specific); high-lard diet altered 7 (weaning), 82 (early adulthood), 3 (LN-specific), and 6 (LH-specific) DEGs, respectively. No persistent DEGs across stages were found. Key DEGs: 9 PN-specific (e.g., *Adam7*, *Ltf*, *Ugt1a5*) (Figure 5b, Supplementary Table S10, DEGs) and 2 LN-specific (*Cfd*, *Gm7375*) (Figure 5c, Supplementary Table S11, DEGs) in testes; 6 LH-specific (e.g., *Slc24a5*) (Figure 5c, Supplementary Table S12, DEGs). In females, ovarian DEGs for the maternal high-palm-oil or -lard group were 8/11 (weaning), 1/7 (early adulthood), 113/8 (late adulthood), and 3/19 (HFD re-exposure), with no persistent DEGs. Key DEGs: 113 PN-specific (Figure 5d, Supplementary Table S13, DEGs) and 18 LH-specific (Figure 5e, Supplementary Table S14, DEGs). GO/KEGG analyses revealed that in male testes, PN-enriched genes (e.g., *Ltf*, *Ugt1a5*) were involved in steroid metabolism, glucuronidation, and pathways like steroid hormone biosynthesis (Figure 5f, Supplementary Tables S15 and S16, GO/KEGG), while LH-enriched *Slc24a5* was associated with melanin metabolism, cation transport, and Golgi-related components (Figure 5g, Supplementary Table S17, GO). In female ovaries, PN-enriched genes (e.g., *Pde2a*, *Vcl*, *Nedd4*, *Atp7a*) were enriched in membrane rafts, ubiquitin binding, and ion binding (Figure 5h, Supplementary Table S18, GO), whereas LH-enriched genes (e.g., *Aqp2*, *Ptgs2*) participated in ovulation, sodium homeostasis, and apoptotic peptidase inhibition (Figure 5i, Supplementary Tables S19 and S20, GO/KEGG). Results indicate maternal saturated fat intake exerts distinct, stage-specific transcriptomic effects on offspring gonads with significant sexual dimorphism. PPI network analysis revealed connections between our identified proteins and key external partners: In male PN, UGT1A5 interacted with the steroid-metabolizing enzymes CYP1B1 and CYP1A1 (Figure 5j). In male LH, SLC24A5 linked to the apoptosis regulator EDAR (Figure 5k). In female PN, NEDD4 associated with PMEPA1 (a TGF-β negative regulator) and the ubiquitination-related protein ARRDC3 (Figure 5l). No significant interactors of LAMC3 were identified in the female LH group (Figure 5m). Consistent with the RNA-seq findings, qPCR analysis confirmed significant upregulation of *Ugt1a5* in PN testes (Figure 5n) and elevated expression of *Slc24a5* in LH testes (Figure 5o). *Nedd4* was downregulated in PN ovaries (Figure 5p), whereas *Lamc3* exhibited increased expression in LH ovaries (Figure 5q).

## 4. Discussion

This study revealed the distinct effects of excessive maternal palm oil or lard intake during pregnancy and lactation on offspring reproductive function, demonstrating notable sex-specific differences and dynamic changes across several developmental stages. Excessive consumption of palm oil or lard impairs reproductive function in offspring of both sexes: reproductive damage induced by maternal high palm-oil exposure is largely independent of the offspring’s postnatal diet, whereas the detrimental effects of maternal high lard exposure are predominantly aggravated by a postnatal high-fat diet in offspring.

Sperm quality (e.g., motility and malformation rate) is a key indicator of male reproductive capacity [22,23]. Impaired sperm quality markedly elevates risks of male infertility and congenital malformations in offspring [24,25]. While previous studies have reported that maternal high-saturated-fat diets reduce sperm motility and increase sperm malformation rate in progeny [26,27], our study reveals divergent effects of distinct saturated fat types on offspring sperm quality. Excessive maternal lard intake significantly increases susceptibility of sperm malformation to HFD rechallenge during adulthood in male offspring, whereas excess palm oil does not induce such effects. Histological analysis indicates that maternal overconsumption of lard causes more pronounced reductions in seminiferous tubule diameter and spermatogenic epithelium thickness in male offspring; these alterations are not observed in the palm oil group.

To further explore the underlying mechanisms, we detected oxidative stress, proliferation, and apoptosis. However, no significant alterations in these processes were observed under the HFD re-exposure condition, particularly in the maternal high-lard group. GO functional enrichment analysis was performed on genes specifically expressed in the LH group, and only *Slc24a5* was enriched. *Slc24a5* is closely associated with metal ion transmembrane transporter activity and cellular calcium ion homeostasis. Previous studies have demonstrated that *Slc24a5* acts as a Na^+^/K^+^/Ca^2+^ transporter [28]. Based on these, *Slc24a5* might be associated with HFD re-exposure-exacerbated sperm impairment induced by excessive maternal lard intake.

Additionally, under normal adult diets, reduced sperm motility resulting from maternal excess lard may be mediated by apoptosis, whereas that induced by maternal high palm oil likely arises from suppressed proliferation and thinner spermatogenic epithelium. *Ugt1a5* was specifically upregulated in the PN group, and GO enrichment analysis revealed its primary involvement in steroid hormone metabolism and synthesis. *Ugt1a5* expression is tightly linked to testosterone glucuronidation activity regulated by hormonal signaling [29,30]. We observed significantly lower serum testosterone and FSH concentrations in the PN group. Therefore, excessive maternal palm oil intake-induced *Ugt1a5* overexpression might be associated with testicular hypoplasia and ultimately impair male reproductive function.

In female offspring, excessive maternal palm oil intake during pregnancy and lactation significantly reduced the primordial follicle count and increased the follicular atresia rate in late adulthood. In contrast, only maternal high-lard intake in early life exhibited increased follicular atresia when re-exposed to an HFD in adulthood. To clarify the mechanisms of this specific damage induced by high-palm-oil exposure, we first measured ovarian index and sex hormones, showing reduced ovarian index and FSH. We also observed inhibited ovarian cell proliferation without altered apoptosis, suggesting decreased primordial follicles in adult offspring may result from impaired proliferation. However, maternal high-lard intake also lowered FSH levels and ovarian proliferation, indicating current results cannot fully explain palm oil’s specific effects. GO enrichment analysis revealed that *Nedd4* was tightly linked to membrane rafts and microdomains. Previous studies have demonstrated that downregulated *Nedd4* impairs granulosa cell function, disrupting bidirectional oocyte-granulosa cell communication [31]. This disruption limits trophic signaling to oocytes and contributes to oocyte dysplasia and follicular atresia [32,33]. Accordingly, suppressed ovarian *Nedd4* expression induced by maternal high-palm-oil exposure might be associated with increasing follicular atresia in female offspring.

Upon the same measurements of oxidative stress, apoptosis, and proliferation in male offspring, we found that a second HFD challenge markedly upregulated antioxidant enzymes in female offspring exposed to a maternal high-lard diet. However, no changes in ovarian cell proliferation or apoptosis were detected. Transcriptomic analysis further identified significant alterations in *Lamc3* expression. Aberrant *Lamc3* overexpression disrupts normal assembly of the follicular basement membrane and impairs granulosa cell survival signaling through integrins [34,35]. These data indicate that follicular atresia in female offspring with maternal high-lard exposure following HFD rechallenge arises from excessive oxidative stress, probably attributed to dysregulated *Lamc3*.

Although excessive maternal intake of saturated fats from different sources exerts differential effects on offspring gonadal function, offspring of different genders exhibit varying sensitivities to the damage induced by the same maternal HFD. For instance, female offspring are more sensitive to oxidative stress damage caused by maternal high-saturated-fat diets than male offspring, which is observable both in the juvenile period and after HFD rechallenge in late adulthood, but not in male testes. Meanwhile, ovarian cell proliferation in female offspring is significantly inhibited by both maternal HFDs from the juvenile period onwards, and this inhibition persists into adulthood. In contrast, male offspring only show proliferation inhibition in the maternal high-palm-oil group, and this phenomenon occurs only in late adulthood. However, gonadal cell apoptosis in offspring induced by excessive lard intake during pregnancy and lactation is more likely to be observed in male offspring. These results also suggest that individualized prevention and control strategies should be implemented for offspring of different genders following maternal HFD exposure.

This study has several limitations. First, future studies are required to identify the upstream factors driving the dysregulation of *Ugt1a5*, *Slc24a5*, *Nedd4*, and *Lamc3*, and to verify their changes in protein expression, as well as whether these molecular changes mediate offspring reproductive dysfunction induced by maternal high-saturated-fat diets with different fat sources. Second, the limited biological replicates of RNA-seq analysis may compromise the accuracy of predictive results. Third, this study lacks direct functional fertility outcomes; further investigation is warranted to explore the direct impacts of maternal overconsumption of different saturated fats on offspring fertility performance, including conception rate, litter size, and postnatal survival rate. Fourth, the currently identified genes are only candidate regulators, rather than definitive drivers, of reproductive dysfunction in offspring. Furthermore, the involvement of aberrant fatty acid metabolism in this process requires further validation. Protective dietary interventions such as intermittent fasting and time-restricted feeding are well established to effectively alleviate HFD-induced oxidative stress, reduce lipid accumulation, and restore metabolic homeostasis [36,37]. Accordingly, we recommend that future studies incorporate these dietary interventions into personalized therapeutic strategies to examine their capacity to mitigate transcriptomic and epigenetic damage triggered by maternal overconsumption of saturated fats, thereby enhancing the translational relevance of our findings in reproductive and metabolic health research.

## 5. Conclusions

In summary, this study reveals that excessive maternal intake of palm oil or lard during pregnancy and lactation impairs reproductive function in a sex- and source-specific manner via distinct mechanisms. This work provides novel insights into nutritional interventions during pregnancy and lactation.

## Data Availability

The raw FASTQ sequencing data and processed datasets, including differentially expressed gene lists, have been deposited in the GEO database, ACCESSION number: GSE341983. The reviewer link: https://www.ncbi.nlm.nih.gov/geo/query/acc.cgi?acc=GSE341983 (secure token: kdaluueynzkhpof) (accessed on 31 July 2026).

## Supplementary Materials

The following supporting information can be downloaded at: https://www.mdpi.com/article/10.3390/nu18162631/s1, Figure S1: Venn diagram of stage-specific DEGs screening; Supplementary Table S1. Diet formula composition of diets used during gestation and lactation; Supplementary Table S2. Diet formula composition of diets used during HFD re-exposure; Supplementary Table S3. Fatty acid composition of diets (g/100g) used during gestation and lactation; Supplementary Table S4. Fatty acid composition of diets (g/100g) used during HFD re-exposure; Supplementary Table S5. Primer sequences used for qPCR; Supplementary Table S6. Serum FFA profile of male offspring after re-exposure to HFD in their adult life; Supplementary Table S7. Serum FFA profile of female offspring after re-exposure to HFD in their adult life; Supplementary Table S8. Changes in serum lipid of male offspring after re-exposure to HFD in their adult life; Supplementary Table S9. Changes in serum lipid of female offspring after re-exposure to HFD in their adult life; Supplementary Table S10. Differentially expressed genes in the testis specific to the high-palm-oil group of male offspring after re-exposure to a normal diet in their adult life; Supplementary Table S11. Differentially expressed genes in the testis specific to the high-lard group of male offspring after re-exposure to a normal diet in their adult life; Supplementary Table S12. Differentially expressed genes in the testis specific to the high-lard group of male offspring after re-exposure to HFD in their adult life; Supplementary Table S13. Differentially expressed genes in the testis specific to the high-palm-oil group of female offspring after re-exposure to a normal diet in their adult life; Supplementary Table S14. Differentially expressed genes in the testis specific to the high-lard group of female offspring after re-exposure to HFD in their adult life; Supplementary Table S15. Results of Gene Ontology (GO) biological-process enrichment analysis for male PN groups; Supplementary Table S16. Results of Kyoto Encyclopedia of Genes and Genomes (KEGG) pathway enrichment analysis for male PN groups; Supplementary Table S17. Results of Gene Ontology (GO) biological-process enrichment analysis for male LH groups; Supplementary Table S18. Results of Gene Ontology (GO) biological-process enrichment analysis for female PN groups; Supplementary Table S19. Results of Gene Ontology (GO) biological-process enrichment analysis for female LH groups; Supplementary Table S20. Results of Kyoto Encyclopedia of Genes and Genomes (KEGG) pathway enrichment analysis for female LH groups.

## Abbreviations

The following abbreviations are used in this manuscript:

E_2_	17β-estradiol
FSH	Follicle-stimulating hormone
GPX	Glutathione peroxidase
HFD	High-fat diet
SOD	Superoxide dismutase
TT	Total Testosterone

## References

[1] Zheng L., Xu X., Zhou J.-Z., Hong L., He Y.-F., Fang Y.-X., Wang B.-B., Chen H., Chen K.-J., Yang S.-S. (2025). The burden of polycystic ovary syndrome-related infertility in 204 countries and territories, 1990–2021: An analysis of the global burden of disease study 2021. Front. Endocrinol..

[2] Zeng G., Liu L., Wang Y., Yu J., Wang H., Li F. (2025). Global, regional, and national burden and trends of reproductive-aged male and female infertility from 1990–2021. Front. Endocrinol..

[3] Luo X., Yin C., Shi Y., Du C., Pan X. (2023). Global trends in semen quality of young men: A systematic review and regression analysis. J. Assist. Reprod. Genet..

[4] Szabó A., Nyirády P., Kopa Z. (2025). Impact of lifestyle and environmental factors on fertility. Curr. Opin. Urol..

[5] Paula V.G., Vesentini G., Sinzato Y.K., Moraes-Souza R.Q., Volpato G.T., Damasceno D.C. (2022). Intergenerational high-fat diet impairs ovarian follicular development in rodents: A systematic review and meta-analysis. Nutr. Rev..

[6] Di Berardino C., Peserico A., Capacchietti G., Zappacosta A., Bernabò N., Russo V., Mauro A., El Khatib M., Gonnella F., Konstantinidou F. (2022). High-Fat Diet and Female Fertility across Lifespan: A Comparative Lesson from Mammal Models. Nutrients.

[7] Wirawan F., Yudhantari D.G.A., Gayatri A. (2023). Pre-pregnancy Diet to Maternal and Child Health Outcome: A Scoping Review of Current Evidence. J. Prev. Med. Public Health.

[8] Gawlińska K., Gawliński D., Filip M., Przegaliński E. (2021). Relationship of maternal high-fat diet during pregnancy and lactation to offspring health. Nutr. Rev..

[9] Williams L., Seki Y., Vuguin P.M., Charron M.J. (2014). Animal models of in utero exposure to a high fat diet: A review. Biochim. Biophys. Acta Mol. Basis Dis..

[10] Zong G., Li Y., Wanders A.J., Alssema M., Zock P.L., Willett W.C., Hu F.B., Sun Q. (2016). Intake of individual saturated fatty acids and risk of coronary heart disease in US men and women: Two prospective longitudinal cohort studies. BMJ.

[11] Bujo S., Toko H., Ito K., Koyama S., Ishizuka M., Umei M., Yanagisawa-Murakami H., Guo J., Zhai B., Zhao C. (2023). Low-carbohydrate diets containing plant-derived fat but not animal-derived fat ameliorate heart failure. Sci. Rep..

[12] Koontanatechanon A., Wongphatcharachai M., Nonthabenjawan N., Jariyahatthakij P., Khorporn T., Parnsen W., Keattisin B., Leksrisompong P., Srichana P., Prasopdee S. (2022). Effects of Omega-3-Rich Pork Lard on Serum Lipid Profile and Gut Microbiome in C57BL/6NJ Mice. Int. J. Food Sci..

[13] Wong G., Clegg M.E., Ross D., Lovegrove J.A., Jackson K.G. (2025). Sequential Meals Containing Animal and Plant-Based Saturated Fats Have Differential Effects on Postprandial Gut Hormones but No Impact on Satiety Compared with Unsaturated Fats in Generally Healthy Males: Findings from the Randomized Controlled Crossover CocoHeart Study. J. Nutr..

[14] Barreiro A., Del Carpio A., Rodríguez-Ferreiro J., Barberia I. (2025). Presentation format influences the strength of causal illusions. Mem. Cogn..

[15] Zhu C., Li P., Li Y., Jiang Y., Liu D., Luo W. (2022). Implicit emotion regulation improves arithmetic performance: An ERP study. Cogn. Affect. Behav. Neurosci..

[16] Ji F., Sadreyev R.I. (2018). RNA-seq: Basic Bioinformatics Analysis. Curr. Protoc. Mol. Biol..

[17] Conesa A., Madrigal P., Tarazona S., Gomez-Cabrero D., Cervera A., McPherson A., Szcześniak M.W., Gaffney D.J., Elo L.L., Zhang X. (2016). A survey of best practices for RNA-seq data analysis. Genome Biol..

[18] Ukeda H., Kawana D., Maeda S., Sawamura M. (1999). Spectrophotometric Assay for Superoxide Dismutase Based on the Reduction of Highly Water-soluble Tetrazolium Salts by Xanthine-Xanthine Oxidase. Biosci. Biotechnol. Biochem..

[19] Deng C., Zhou Q., Zhang M., Li T., Chen H., Xu C., Feng Q., Wang X., Yin F., Cheng Y. (2022). Bioceramic Scaffolds with Antioxidative Functions for ROS Scavenging and Osteochondral Regeneration. Adv. Sci..

[20] Ellman G.L. (1959). Tissue Sulfhydryl Groups. Arch. Biochem. Biophys..

[21] Peng H., Zhu M., Kong W., Tang C., Du J., Huang Y., Jin H. (2023). L-cystathionine protects against oxidative stress and DNA damage induced by oxidized low-density lipoprotein in THP-1-derived macrophages. Front. Pharmacol..

[22] Pan M., Li J., Wang Y., Liu Z., Li L., Wang T. (2025). The downregulation of hormone-sensitive lipase and dysregulation of cholesterol receptors/transporter affect testicular lipid homeostasis and function in HFD-induced oligoasthenospermia mice. Mol. Med..

[23] Su M., Gan S., Gao R., Du C., Wei C., Shah A.M., Ma J. (2025). Toxicity Mechanisms of Microplastic and Its Effects on Ruminant Production: A Review. Biomolecules.

[24] Mashayekh-Amiri S., Asghari Jafarabadi M., Molaie B., Rashidi F., Hemati E., Aliasghari F., Mirghafourvand M. (2024). Validation and measurement properties of the Male and Female Fertility Knowledge Inventories (MFKI and FFKI) in Iranian couples. Reprod. Health.

[25] Yadav A., Tiwari P., Kumar R., Dada R. (2025). Impact of a 12-week Yoga Intervention on Seminal Oxidative Stress, Sperm Quality, and DNA Fragmentation Index in Infertile Men: A Pre-post Intervention Study. Int. J. Yoga.

[26] Qi X., Guan Q., Zhang W., Huang X., Yu C. (2023). The time-dependent adverse effects of a high-fat diet on sperm parameters. Adv. Clin. Exp. Med..

[27] Meneghini M.A., Galarza R.A., Flores Quiroga J.P., Faletti A.G. (2022). Diet-induced maternal obesity and overnutrition cause a decrease in the sperm quality of the offspring. J. Nutr. Biochem..

[28] Ginger R.S., Askew S.E., Ogborne R.M., Wilson S., Ferdinando D., Dadd T., Smith A.M., Kazi S., Szerencsei R.T., Winkfein R.J. (2008). SLC24A5 Encodes a *trans*-Golgi Network Protein with Potassium-dependent Sodium-Calcium Exchange Activity That Regulates Human Epidermal Melanogenesis. J. Biol. Chem..

[29] Buckley D.B., Klaassen C.D. (2009). Mechanism of gender-divergent UDP-glucuronosyltransferase mRNA expression in mouse liver and kidney. Drug Metab. Dispos..

[30] Neumann E., Mehboob H., Ramírez J., Mirkov S., Zhang M., Liu W. (2016). Age-Dependent Hepatic UDP-Glucuronosyltransferase Gene Expression and Activity in Children. Front. Pharmacol..

[31] Fang W.L., Lai S.Y., Lai W.A., Lee M.T., Liao C.F., Ke F.C., Hwang J.J. (2015). CRTC2 and Nedd4 ligase involvement in FSH and TGFβ1 upregulation of connexin43 gap junction. J. Mol. Endocrinol..

[32] Windley S.P., Mayère C., McGovern A.E., Harvey N.L., Nef S., Schwarz Q., Kumar S., Wilhelm D. (2022). Loss of NEDD4 causes complete XY gonadal sex reversal in mice. Cell Death Dis..

[33] Matsuda F., Inoue N., Manabe N., Ohkura S. (2012). Follicular growth and atresia in mammalian ovaries: Regulation by survival and death of granulosa cells. J. Reprod. Dev..

[34] Irving-Rodgers H.F., Rodgers R.J. (2005). Extracellular matrix in ovarian follicular development and disease. Cell Tissue Res..

[35] Tanimoto R., Yoshida K., Ikeda S., Obata Y. (2022). Insights into in vivo follicle formation: A review of in vitro systems. Histochem. Cell Biol..

[36] Diab R., Dimachkie L., Zein O., Dakroub A., Eid A.H. (2024). Intermittent Fasting Regulates Metabolic Homeostasis and Improves Cardiovascular Health. Cell Biochem. Biophys..

[37] Chen Y., Su J., Yan Y., Zhao Q., Ma J., Zhu M., He X., Zhang B., Xu H., Yang X. (2021). Intermittent Fasting Inhibits High-Fat Diet–Induced Atherosclerosis by Ameliorating Hypercholesterolemia and Reducing Monocyte Chemoattraction. Front. Pharmacol..

