# Supplementary material for: Maternal Excessive Lard Versus Palm Oil Intake in Mice Drives Sex-Specific Reproductive Dysfunction in Offspring
Source: Nutrients. 2026 Aug 12;18(16):2631. doi: 10.3390/nu18162631 (PMC13516037; doi:10.3390/nu18162631)
Supplement: Supplementary file 1 [file nutrients-18-02631-s001.zip › nutrients-4441833-supplementary.pdf]

Maternal excessive lard *versus* palm oil intake in mice drives sex-specific reproductive dysfunction in offspring
Yan Xu

Supplementary Table S1. Diet formula composition of diets used during gestation and lactation

**Ingredients (g/kg）**	**CD**	**HP**	**HL**
Product	SY93G	SYHPA60	SYHSA60
Casein	200	240	240
L-Cystine	3	4	4
Corn Starch	397.5	0	0
Maltodextrin	132	183.6	183.6
Sucrose	100	100	100
cellulose	50	56	56
Soybean oil	70	40	40
Palm Oil	0	314	0
Lard	0	0	314
Mineral MIX S10022G	35	46	46
VitaminMixV10037	10	13	13
Choline Bitartrate	2.5	3.4	3.4
FD＆Dye 1	0	0.05	0.05
Total energy (kcal/kg)	4000	5350	5350
Fat energy ratio (%)	15.75	60	60
Carbohydrate energy ratio (%)	63.95	22	22
Protein energy ratio (%)	20.3	18	18

CD, control diet (15.75% energy from soybean oil, SY93G); HP, high-palm oil diet (60% energy from palm oil, SYHPA60); HL, high-lard diet (60% energy from lard, SYHSA60). The diets during gestation and lactation were purchased from Shuyu Biotech, Shanghai, China.

Supplementary Table S2. Diet formula composition of diets used during HFD re-exposure

**Ingredients (g/kg）**	**ND**	**HFD**
Product	H10010	H10045
Casein	189.58	233.06
L-Cystine	2.84	3.5
Corn Starch	298.59	84.83
Maltodextrin	33.18	116.53
Sucrose	331.77	201.36
Cellulose	47.4	58.26
Soybean oil	23.7	29.13
Palm Oil	0	0
Lard	18.96	206.84
Mineral MIX M1002	9.48	11.65
VitaminMix V1001	9.48	11.56
Choline Bitartrate	1.9	2.33
Dicalcium Phosphate	12.32	15.15
Calcium Carbonate	5.21	6.41
Potassium Citrate	15.64	19.23
FD＆Dye 1	0.047	0.058
Total energy (kcal/kg)	3850	4730
Fat energy ratio (%)	10	45
Carbohydrate energy ratio (%)	70	35
Protein energy ratio (%)	20	20

ND, normal diet (10% energy from fat, H10010); HFD, High-fat diet (45% energy from fat, H10045). The diets for the high-fat diet rechallenge in adulthood were supplied by Beijing HFK Bioscience Co., Ltd.

Supplementary Table S3. Fatty acid composition of diets (g/100g) used during gestation and lactation

FFAs (g/100g)	CD	HP	HL
Palmitic acid (C16:0, PA)	1.66	23.33	9.82
Palmitoleic acid (C16:1, PLA)	0.17	2.02	0.75
Stearic acid (C18:0, SA)	0.68	3.36	5.91
Oleic acid (C18:1, OA)	0.5	2.6	4.27
Linoleic acid (C18:2, LA)	4.61	3.91	7.29
α-Linolenic acid (α-C18:3, α-LNA)	0.01	0.01	0.01
Arachidonic acid (C20:4, AA)	0.34	2.97	3.57
Saturated fatty acids	2.35	26.69	15.72
Stearic acid percentage (%)	8.49	8.8	18.68
Palmitic acid percentage (%)	21	61.05	31.03
Stearic-to-palmitic acid ratio	0.41	0.14	0.6
Unsaturated fatty acids	5.62	11.51	15.89
ω-3 fatty acids	0.01	0.01	0.01
ω-6 fatty acids	4.61	3.91	7.29
Total fatty acids	7.97	38.2	31.61

Data are presented as weight percentages of total fatty acids. CD, control diet (15.75% energy from soybean oil, SY93G); HP, high-palm oil diet (60% energy from palm oil, SYHPA60); HL, high-lard diet (60% energy from lard, SYHSA60). The diets during gestation and lactation were purchased from Shuyu Biotech, Shanghai, China.

Supplementary Table S4. Fatty acid composition of diets (g/100g) used during HFD re-exposure

FFAs (g/100g)	ND	HFD
Palmitic acid (C16:0, PA)	2.89	9.4
Palmitoleic acid (C16:1, PLA)	0.27	0.8
α-Linolenic acid (α-C18:3, α-LNA)	0.01	0.01
Arachidonic acid (C20:4, AA)	1.02	2.85
Stearic acid (C18:0, SA)	1.14	4.86
Oleic acid (C18:1, OA)	0.84	3.5
Linoleic acid (C18:2, LA)	5.61	4.41
Saturated fatty acids	4.02	14.26
Stearic acid percentage (%)	9.6	18.83
Palmitic acid percentage (%)	24.53	36.35
Stearic-to-palmitic acid ratio	0.39	0.52
Unsaturated fatty acids	7.75	11.57
ω-3 fatty acids	0.01	0.01
ω-6 fatty acids	6.63	7.26
Total fatty acids	11.78	25.83

Values are expressed as weight percentage of total fatty acids. ND, normal diet (10% energy from fat, H10010); HFD, High-fat diet (45% energy from fat, H10045). The diets for the High-Fat Diet Challenge in Late Adulthood were supplied by Beijing HFK Bioscience Co., Ltd.

Supplementary Table S5. Primer sequences used for qPCR

Gene	Primer	Primer sequence (5'-3')	Product size (bp)
Ugt1a5	Forward	ATCACTCCCTATGAAAGCCTG	80
	Reverse	ACGGATGCATAGCTGAAAAGC	
Nedd4	Forward	CAAGAGCACACACCTGCTTCA	117
	Reverse	AATCAACGCCATCAAAGCCC	
Slc24a5	Forward	GAAATGCGGACAAAATGGGGC	139
	Reverse	ATTGCGTGCTGTTTCCTGTGG	
Lamc3	Forward	TGTGCAACCATCCCAGAGAGTG	77
	Reverse	CTCTCGCATCGTCGTCCTCT	

Note: All primer sequences are presented in the 5′ to 3′ orientation. Product size indicates the length of amplified fragments.

Supplementary Table S6. Serum FFA profile of male-offspring after re-exposure to HFD in their adult life.

FFAs (μg/ml)	Effect of diet	CN (n=10)	PN (n=10)	LN (n=10)	CH (n=10)	PH (n=10)	LH (n=10)
Myristic acid (C14:0, MA)	RE	1.45±0.19	1.4±0.14	1.52±0.19	0.59±0.08***	0.93±0.12*	1±0.07*
Palmitic acid (C16:0, PA)	RE	390.03±19.01	380.81±30.63	387.87±29.63	503.55±14.2*	516.55±25.59*	668.39±78.02$^{***\#\wedge}$
Palmitoleic acid (C16:1, PLA)	RE	46.68±1.6	45.94±2.34	50.52±2.6	66.32±5.16*	66.72±2.15*	88.61±12.74$^{***\#\wedge}$
Stearic acid (C18:0, SA)	RE	119.93±12.12	115.43±8.9	127.13±27.8	219.37±17.31*	255.93±7.74**	371.36±74$^{***\#\#}$
Oleic acid (C18:1, OA)	RE	114.07±11.86	104.92±12.56	109.53±20.03	192.7±5.1*	251.15±38.16***	299.7±47.84$^{***\#}$
Linoleic acid (C18:2, LA)	RE	525.47±44.45	526.99±20.69	522.64±37.02	402.84±14.4**	409.94±24.71**	406.02±20.08**
γ-Linolenic acid (γ-C18:3, γ-LNA)	-	9.91±0.97	9.19±0.59	8.49±0.54	9.23±0.91	9.32±0.87	11.2±0.52*
α-Linolenic acid (α-C18:3, α-LNA)	RE	0.38±0.04	0.38±0.03	0.38±0.02	0.17±0.01***	0.17±0.01***	0.2±0.01***
Eicosatrienoic acid (C20:3, EA)	RE	21.68±1.48	16.13±0.68	25.26±4.61	54.67±6.85***	56.39±7.27***	48.05±3.36**
Arachidonic acid (C20:4, AA)	RE	468.06±37.5	404.62±8.75	453.84±38.85	1246.7±96.69***	1272±130.78***	1318.12±99.78***
Docosapentaenoic acid (C22: 5, DPA)	-	13.3±1.29	11.15±0.59	14.81±1.52	15.62±1.22	13.87±1.57	15.02±2.23
Docosahexaenoic acid (C22:6, DHA)	RE	609.99±50.61	534.37±26.96	617.43±58.34	1097.47±80.07***	1043.75±92.24***	1081.38±114.54***
Saturated fatty acids	RE	540.02±45.41	503.77±50.61	504.66±56.54	767.37±87.87*	754.97±25.64*	1053.57±146.22$^{***\#\wedge}$
Stearic acid percentage (%)	GLxRE,RE	6.61±0.61	5.18±0.37	5.36±0.75	5.86±0.4	6.72±0.49	8.73±1.47$^{**\#}$
Palmitic acid percentage (%)	RE	17.46±0.9	17.64±0.79	17.3±0.75	12.55±0.69***	12.94±0.57***	14.45±1.17*
Stearic-to-palmitic acid ratio	RE	0.34±0.03	0.3±0.02	0.32±0.04	0.44±0.03*	0.48±0.03***	0.54±0.06***
Unsaturated fatty acids	RE	1804.82±121.92	1674.8±66.42	1827.09±105.18	3066.55±185.09***	3000.76±206.81***	3023.07±268.23***
ω-3 fatty acids	RE	640.54±52.96	613.13±30.35	628.23±65.11	1152.46±77.34***	1180.62±88.04***	1121.13±118.2***
ω-6 fatty acids	RE	1000.75±72.36	934.96±48.5	984.25±67.67	1618.65±112.09***	1650.83±138.64***	1670.2±141.55***
Total fatty acids	RE	2365.37±150.9	2172.21±113.41	2269.31±185.04	3876.22±239.44***	3672.15±296.53***	3927.23±311.72***

$^{*}P < 0.05$, $^{**}P < 0.01$, $^{***}P < 0.001$ *vs* CN, PN or LN, respectively.$^{\#}$P < 0.05, $^{\#\#}P < 0.01$ vs CH; $^{\wedge}P < 0.05$ vs PH. CN, PN, LN, first exposure to CD/HP/HL and re-exposure to normal diet; CH, PH, LH, first exposure to CD/HP/HL and re-exposure to high-fat diet. HFD, High-fat diet of 45% energy from fat. GL: effect of HFD exposure during gestation and lactation; RE: effect of HFD re-exposure; GL×RE: interactive effect of GL and RE.

Maternal excessive lard *versus* palm oil intake in mice drives sex-specific reproductive dysfunction in offspring
Yan Xu

Supplementary Table S7. Serum FFA profile of female-offspring after re-exposure to HFD in their adult life.

FFAs (μg/ml)	Effect of diet	CN (n=10)	PN (n=10)	LN (n=10)	CH (n=10)	PH (n=10)	LH (n=10)
Myristic acid (C14:0, MA)	-	1.48±0.28	1.41±0.18	1.13±0.16	1.15±0.2	1±0.1	1.13±0.08
Palmitic acid (C16:0, PA)	-	294.32±42.8	295.01±16.1	297.98±19.63	283.53±24.32	303.57±23.55	305.62±39.03
Palmitoleic acid (C16:1, PLA)	-	40.09±4.19	39.54±1.72	37.56±2.29	40.8±1.8	38.99±2.66	41.71±3.19
Stearic acid (C18:0, SA)	-	110.83±10.62	116.96±12.72	113.51±14.85	144.65±12.97	119.53±20.16	145.03±18.99
Oleic acid (C18:1, OA)	-	106.32±13.03	90.15±9.58	89.44±10.82	115.91±8.91	109.38±13.33	101.52±8.39
Linoleic acid (C18:2, LA)	RE	348.97±36.2	390.87±27.09	337.38±26.63	235.57±17.39**	185.33±22.98***	224.87±18.44**
γ-Linolenic acid (γ-C18:3, γ-LNA)	-	6.75±0.86	7.07±0.63	6.43±0.51	6.07±0.37	6.36±0.61	6.2±0.55
α-Linolenic acid (α-C18:3, α-LNA)	GLxRE, RE	0.23±0.03	0.26±0.03	0.33±0.03*	0.11±0.01***	0.12±0.01***	0.1±0.01***
Eicosatrienoic acid (C20:3, EA)	RE	9.51±0.99	9.38±0.65	9.85±0.93	15.51±1.52**	16.07±2.29**	18.82±1.68***
Arachidonic acid (C20:4, AA)	RE	397.2±32.5	379.07±31.01	342.23±33.51	681.11±61.32***	602.09±92.3**	630.21±64.46***
Docosapentaenoic acid (C22: 5, DPA)	RE	7.41±0.61	6.69±0.67	7.52±0.96	4.86±0.34*	5.65±0.85	4.82±0.61**
Docosahexaenoic acid (C22:6, DHA)	-	458.15±40.09	431.11±31.91	486.23±44.46	518.06±65	515.19±115.81	505.11±62.23
Saturated fatty acids	-	1028.1±117.41	1000.72±161.78	1122.14±144.96	1125.56±137.66	1093.91±147.31	1324.91±75.54
Stearic acid percentage (%)	-	7.21±0.62	6.41±0.48	6.51±0.4	6.8±0.36	6.81±0.53	6.95±0.25
Palmitic acid percentage (%)	-	15.24±1.34	16.83±0.38	16.45±0.7	14.83±0.67	17.36±1.09	15.82±1.13
Stearic-to-palmitic acid ratio	RE	0.48±0.05	0.35±0.03	0.4±0.03	0.48±0.03	0.49±0.05*	0.51±0.02*
Unsaturated fatty acids	-	1328.62±110.42	1374.64±100.58	1335.76±105.49	1499.18±156.86	1329.39±243.23	1425.96±135.04
ω-3 fatty acids	-	474.83±41.52	453.67±48.32	459.98±55.31	546.25±62.46	533.99±118.46	517.7±66.92
ω-6 fatty acids	-	742.19±63.14	748.66±60.85	722.39±61.09	822.27±78.55	759.33±108.54	869.11±72.15
Total fatty acids	-	1801.77±159.03	1773.44±126.68	1775.3±138.67	2040.53±218.4	1920.27±271.74	2058.54±185.19

*P < 0.05, **P < 0.01, ***P < 0.001 vs CN, PN or LN, respectively. CN, PN, LN, first exposure to CD/HP/HL and re-exposure to normal diet; CH, PH, LH, first exposure to CD/HP/HL and re-exposure to high-fat diet. HFD, High-fat diet of 45% energy from fat. GL: effect of HFD exposure during gestation and lactation; RE: effect of HFD re-exposure; GL×RE: interactive effect of GL and RE.

Supplementary Table S8. Changes in serum lipid of male-offspring after re-exposure to HFD in their adult life.

Serum lipid parameters	Effect of diet	CN (n=10)	PN (n=10)	LN (n=10)	CH (n=10)	PH (n=10)	LH (n=10)
Total Cholesterol (mg/dl)	RE	3.02±0.03	2.68±0.05	3.11±0.05	6.24±0.38***	6.28±0.32***	6.35±0.17***
Triglycerides (mmol/L)	RE	0.24±0.03	0.3±0.05	0.24±0.02	0.4±0.08*	0.34±0.05	0.41±0.06*
HDL-C (mmol/L)	GL,RE	2.33±0.08	1.66±0.13*	2.11±0.14	4.02±0.22***	3.85±0.22***	3.81±0.16***
LDL-C (mmol/L)	GL×RE, GL,RE	0.51±0.07	0.47±0.05	0.49±0.05	1.43±0.22***	2.29±0.26$^{***\#\#}$	2.23±0.22$^{***\#\#}$
LDL-C/HDL-C	GL,RE	0.2±0.02	0.24±0.02	0.22±0.03	0.38±0.04***	0.51±0.05$^{***\#}$	0.54±0.04$^{***\#\#}$

$^{*}P < 0.05$, $^{**}P < 0.01$, $^{***}P < 0.001$ vs CN, PN or LN, respectively. $^{\#}P < 0.05$, $^{\#\#}P < 0.01$ vs CH. CN, PN, LN, first exposure to CD/HP/HL and re-exposure to normal diet; CH, PH, LH, first exposure to CD/HP/HL and re-exposure to high-fat diet. HFD, High-fat diet of 45% energy from fat. GL: effect of HFD exposure during gestation and lactation; RE: effect of HFD re-exposure; GL×RE: interactive effect of GL and RE.

Supplementary Table S9. Changes in serum lipid of female-offspring after re-exposure to HFD in their adult life.

Serum lipid parameters	Effect of diet	CN (n=10)	PN (n=10)	LN (n=10)	CH (n=10)	PH (n=10)	LH (n=10)
Total Cholesterol (mg/dl)	RE	2.18±0.15	2.11±0.16	2.35±0.1	3.9±0.16***	3.29±0.15$^{***\#}$	3.66±0.22***
Triglycerides (mmol/L)	RE	0.3±0.04	0.31±0.03	0.31±0.04	0.2±0.02*	0.27±0.03	0.18±0.02**
HLD-C (mmol/L)	RE	1.69±0.08	1.65±0.13	1.67±0.12	2.66±0.13***	2.34±0.23***	2.64±0.09***
LDL-C (mmol/L)	GL×RE, GL, RE	0.2±0.02	0.23±0.01	0.23±0.01	0.99±0.09***	0.62±0.09$^{***\#\#}$	1.02±0.13$^{***\wedge\wedge}$
LDL-C/HDL-C	GL×RE, RE	0.12±0.01	0.17±0.02	0.15±0.01	0.38±0.02***	0.26±0.02$^{**\#\#\#}$	0.34±0.03$^{***\wedge}$

$^{*}P < 0.05$, $^{**}P < 0.01$, $^{***}P < 0.001$ vs CN, PN or LN, respectively. $^{\#}P < 0.05$, $^{\#\#}P < 0.01$, $^{\#\#\#}P < 0.001$ vs CH; ^P < 0.05, $^{\wedge\wedge}P < 0.01$ vs PH. CN, PN, LN, first exposure to CD/HP/HL and re-exposure to normal diet; CH, PH, LH, first exposure to CD/HP/HL and re-exposure to high-fat diet. HFD, High-fat diet of 45% energy from fat. GL: effect of HFD exposure during gestation and lactation; RE: effect of HFD re-exposure; GL×RE: interactive effect of GL and RE.

Supplementary Table S10. Differentially expressed genes in the testis specific to the high-palm oil group of male-offspring after re-exposure to a normal diet in their adult life.

Gene ID	Gene name	PN *vs.* CN		PN *vs.* LN	
		Log_2 FC	*P*-value	Log_2 FC	*P*-value
ENSMUSG00000089943	*Ugt1a5*	11.453	6.211E-08	-11.453	8.291E-10
ENSMUSG00000111312	*Gm47205*	1.315	8.716E-03	-1.532	7.979E-06
ENSMUSG00000022056	*Adam7*	-11.741	5.277E-09	11.002	3.745E-09
ENSMUSG00000074679	*Defb28*	-14.056	1.526E-08	13.089	3.109E-08
ENSMUSG00000090785	*Gm17116*	-11.598	4.642E-06	11.645	7.331E-07
ENSMUSG00000096468	*Gm16405*	-1.402	7.871E-03	1.522	1.165E-07
ENSMUSG00000032496	*Ltf*	-9.093	1.385E-02	11.219	4.469E-09
ENSMUSG00000097538	*Gm26939*	-9.272	3.360E-02	12.142	4.358E-09
ENSMUSG00000018211	*Wfdc15b*	-13.472	4.089E-02	12.155	5.665E-08

CN, PN, LN, first exposure to CD/HP/HL and re-exposure to normal diet.

Supplementary Table S11. Differentially expressed genes in the testis specific to the high-lard group of male-offspring after re-exposure to a normal diet in their adult life.

Gene ID	Gene name	LN *vs.* CN		LN *vs.* PN	
		Log_2 FC	*P*-value	Log_2 FC	*P*-value
ENSMUSG00000061780	*Cfd*	14.208	8.031E-10	14.208	7.809E-10
ENSMUSG00000095423	*Gm7375*	13.052	1.203E-08	13.052	1.176E-08

CN, PN, LN, first exposure to CD/HP/HL and re-exposure to normal diet.

Supplementary Table S12. Differentially expressed genes in the testis specific to the high-lard group of male-offspring after re-exposure to HFD in their adult life.

**Gene ID**	**Gene name**	**LH *vs.* CH**		**LH *vs.* PH**	
		**Log_2 FC**	***P*-value**	**Log_2 FC**	***P*-value**
ENSMUSG00000095802	*Gm7415*	16.180	3.180E-09	16.180	2.481E-09
ENSMUSG00000105340	*Gm42878*	12.007	5.794E-09	12.007	4.982E-09
ENSMUSG00000057244	*Gm6139*	1.743	1.677E-06	1.370	1.810E-05
ENSMUSG00000035183	*Slc24a5*	2.078	1.012E-05	2.119	6.987E-06
ENSMUSG00000114184	*Gm7143*	1.519	1.800E-04	1.396	6.356E-06
ENSMUSG00000062554	*Gm12751*	-1.124	4.916E-12	-1.009	3.120E-09

HFD, high-fat diet. CH, PH, LH, first exposure to CD/HP/HL and re-exposure to high-fat diet.

Supplementary Table S13. Differentially expressed genes in the testis specific to the high-palm oil group of female-offspring after re-exposure to a normal diet in their adult life.

Gene ID	Gene name	PN *vs.* CN		PN *vs.* LN	
		Log$_2$ FC	*P*-value	Log$_2$ FC	*P*-value
ENSMUSG00000021133	*Susd6*	-6.640	2.187E-113	6.565	4.757E-156
ENSMUSG00000110195	*Pde2a*	-9.702	1.392E-48	9.467	1.469E-46
ENSMUSG00000064220	*H2ac18*	-6.641	1.614E-16	7.783	7.745E-04
ENSMUSG00000072949	*Acot1*	-1.982	9.896E-15	1.398	4.473E-09
ENSMUSG00000093752	*Gm20716*	-16.085	1.330E-12	17.428	5.195E-13
ENSMUSG00000117098	*Gm49909*	-17.264	6.288E-12	16.519	8.827E-11
ENSMUSG00000107215	*Gm43197*	-12.532	2.902E-10	10.652	7.403E-06
ENSMUSG00000026617	*Bpnt1*	-2.218	7.999E-10	1.832	6.960E-18
ENSMUSG00000102422	*Iqschfp*	13.530	2.686E-09	-13.530	7.994E-10
ENSMUSG00000098950	*Gm28036*	-11.128	9.851E-09	12.151	1.159E-17
ENSMUSG00000028957	*Per3*	1.613	3.679E-08	-1.301	4.326E-10
ENSMUSG00000084911	*Gm16185*	-2.321	3.810E-08	1.792	8.352E-05
ENSMUSG00000048234	*Rnf149*	-2.268	3.825E-08	1.538	4.590E-14
ENSMUSG00000109564	*Muc16*	1.891	4.188E-08	-1.140	8.715E-05
ENSMUSG00000029028	*Lrrc47*	-1.284	7.313E-08	1.155	3.164E-08
ENSMUSG00000006403	*Adamts4*	-3.115	1.019E-07	2.501	4.025E-07
ENSMUSG00000092395	*Gm20463*	13.618	1.044E-07	-13.618	3.223E-08
ENSMUSG00000033792	*Atp7a*	-3.425	1.613E-07	2.226	8.022E-06
ENSMUSG00000064225	*Paqr9*	-1.608	1.776E-07	1.517	5.269E-07
ENSMUSG00000058625	*Gm17383*	12.147	1.915E-07	-12.147	1.963E-08
ENSMUSG00000028989	*Angptl7*	2.426	2.140E-07	-2.390	9.410E-07
ENSMUSG00000044147	*Arf6*	-2.365	5.349E-07	1.698	1.932E-09
ENSMUSG00000021823	*Vcl*	-2.164	5.789E-07	1.371	1.704E-05
ENSMUSG00000025262	*Fam120c*	-1.613	9.155E-07	1.115	7.230E-04
ENSMUSG00000117822	*Eef1a1-ps1*	-1.697	9.169E-07	1.453	5.197E-08
ENSMUSG00000033855	*Ston1*	-2.699	9.467E-07	1.687	4.151E-04
ENSMUSG00000079884	*Gm10698*	-2.367	1.127E-06	1.600	2.960E-06
ENSMUSG00000062563	*Cys1*	2.052	1.265E-06	-1.749	1.917E-07
ENSMUSG00000027940	*Tpm3*	-1.586	1.335E-06	1.072	3.231E-10
ENSMUSG00000044949	*Ubtd2*	-2.569	1.373E-06	1.806	5.924E-06
ENSMUSG00000059195	*Gm12715*	-2.337	1.577E-06	1.612	9.518E-10

ENSMUSG00000024972	*Lgals12*	2.579	2.219E-06	-2.038	3.870E-05
ENSMUSG00000015127	*Unkl*	-1.290	2.609E-06	1.479	3.137E-06
ENSMUSG00000021908	*Ncoa4-ps*	-1.863	2.739E-06	1.427	4.683E-05
ENSMUSG00000081400	*Gm13680*	-1.909	3.414E-06	1.361	9.655E-06
ENSMUSG00000026563	*Tada1*	-1.802	4.016E-06	1.426	5.785E-06
ENSMUSG00000031736	*Crnde*	1.431	4.777E-06	-1.525	2.663E-10
ENSMUSG00000005893	*Nr2c2*	-2.240	5.094E-06	1.213	3.026E-04
ENSMUSG00000026123	*Plekhb2*	-1.667	5.207E-06	1.198	1.386E-05
ENSMUSG00000021774	*Ube2e1*	-1.543	5.319E-06	1.006	1.203E-05
ENSMUSG00000105096	*Gbp10*	-3.108	6.139E-06	3.004	4.074E-04
ENSMUSG00000083287	*Gm13502*	-3.342	7.576E-06	2.780	1.999E-05
ENSMUSG00000034218	*Atm*	-1.684	9.022E-06	1.448	1.282E-04
ENSMUSG00000061724	*Gm2423*	-1.445	9.124E-06	1.159	1.354E-06
ENSMUSG00000021952	*Xpo4*	-1.159	1.102E-05	1.052	2.504E-06
ENSMUSG00000014498	*Ankrd52*	-1.579	1.249E-05	1.314	4.757E-05
ENSMUSG00000034926	*Dhcr24*	-1.739	1.291E-05	1.268	1.588E-08
ENSMUSG00000080902	*Ywhaq-ps3*	-1.470	1.395E-05	1.054	2.406E-05
ENSMUSG00000031352	*Hccs*	-1.371	1.439E-05	1.074	2.847E-06
ENSMUSG00000022667	*Cd200r1*	-2.779	1.645E-05	2.246	4.343E-04
ENSMUSG00000040078	*Ptges3-ps*	-1.623	1.654E-05	1.201	4.129E-06
ENSMUSG00000030263	*Lrmp*	1.491	1.937E-05	-1.524	6.969E-06
ENSMUSG00000022043	*Trim35*	-1.716	2.147E-05	1.178	3.005E-08
ENSMUSG00000047514	*Tspyl1*	-1.566	2.287E-05	1.112	2.318E-05
ENSMUSG00000025739	*Gng13*	1.573	3.745E-05	-1.499	6.701E-04
ENSMUSG00000005580	*Adcy9*	-1.512	4.590E-05	1.154	5.664E-06
ENSMUSG00000020229	*Slc5a4a*	1.737	4.705E-05	-1.854	1.737E-05
ENSMUSG00000116875	*Morf4l1-ps1*	-1.533	4.925E-05	1.223	1.384E-07
ENSMUSG00000005802	*Slc30a4*	-1.565	4.968E-05	1.084	3.225E-06
ENSMUSG00000055116	*Arntl*	-1.600	5.082E-05	1.829	2.338E-06
ENSMUSG00000022257	*Laptm4b*	-1.196	5.306E-05	1.039	1.435E-06
ENSMUSG00000063684	*Hadhb-ps*	-1.527	5.755E-05	1.165	4.206E-06
ENSMUSG00000104156	*Gm38102*	-1.883	6.107E-05	2.111	2.199E-05
ENSMUSG00000041324	*Inhba*	1.423	7.467E-05	-1.437	1.128E-06
ENSMUSG00000099032	*Tcf24*	-1.730	7.558E-05	1.162	1.445E-04
ENSMUSG00000062203	*Gspt1*	-1.461	7.729E-05	1.088	1.040E-04

ENSMUSG00000032112	*Trappc4*	-1.780	7.839E-05	1.293	4.446E-05
ENSMUSG00000024122	*Pdpk1*	-1.363	8.300E-05	1.059	2.960E-06
ENSMUSG00000102976	*Zc3h11a*	-1.456	8.773E-05	2.304	6.218E-04
ENSMUSG00000114940	*Gm21378*	-2.876	8.968E-05	2.422	1.927E-04
ENSMUSG00000032216	*Nedd4*	-1.220	9.098E-05	1.009	7.896E-06
ENSMUSG00000085711	*Gm15163*	-1.538	9.306E-05	1.242	4.031E-04
ENSMUSG00000053522	*Lgals7*	1.205	9.857E-05	-1.123	2.603E-05
ENSMUSG00000104802	*Gm5869*	-1.687	9.892E-05	1.428	3.695E-05
ENSMUSG00000082575	*Eef2-ps2*	-1.240	1.014E-04	1.382	1.082E-06
ENSMUSG00000097504	*4930516B21Rik*	-1.353	1.016E-04	1.236	1.898E-04
ENSMUSG00000100349	*Gm28188*	1.545	1.057E-04	-1.668	2.291E-09
ENSMUSG00000073394	*Runx2os1*	2.894	1.235E-04	-2.856	1.077E-04
ENSMUSG00000043501	*Lgals2*	1.252	1.361E-04	-1.609	5.306E-09
ENSMUSG00000070583	*Fv1*	-2.216	1.413E-04	2.202	7.755E-06
ENSMUSG00000028370	*Pappa*	-2.067	1.468E-04	1.620	1.571E-06
ENSMUSG00000023052	*Npff*	1.927	1.525E-04	-1.035	2.977E-04
ENSMUSG00000039202	*Abhd2*	-1.144	1.537E-04	1.030	2.539E-07
ENSMUSG00000114003	*Gm9616*	-1.360	1.604E-04	1.099	2.364E-05
ENSMUSG00000022389	*Tef*	1.056	1.649E-04	-1.009	8.521E-08
ENSMUSG00000024896	*Minpp1*	-1.357	1.665E-04	1.174	4.795E-06
ENSMUSG00000079645	*Gm17193*	-11.684	1.707E-04	11.512	4.418E-04
ENSMUSG00000079017	*Ifi27l2a*	1.581	1.746E-04	-1.426	1.969E-04
ENSMUSG00000058809	*Hspd1-ps3*	-1.587	1.749E-04	2.138	9.455E-08
ENSMUSG00000042082	*Arsb*	-1.269	1.790E-04	1.003	3.742E-05
ENSMUSG00000095847	*Gm5451*	-1.614	1.958E-04	1.124	3.385E-04
ENSMUSG00000103749	*Pcdhgb5*	-2.785	2.080E-04	3.711	5.194E-07
ENSMUSG00000031403	*Dkc1*	-1.576	2.129E-04	1.230	6.199E-07
ENSMUSG00000115684	*Gm6087*	-1.805	2.146E-04	1.847	1.626E-04
ENSMUSG00000025980	*Hspd1*	-1.017	2.244E-04	1.255	1.704E-05
ENSMUSG00000025870	*Arl10*	-1.608	2.314E-04	1.351	7.096E-07
ENSMUSG00000085337	*Gm15964*	1.491	2.646E-04	-1.066	1.700E-04
ENSMUSG00000035898	*Uba6*	-1.245	2.684E-04	1.016	2.977E-04
ENSMUSG00000037762	*Slc16a9*	-1.219	2.970E-04	1.019	3.552E-06
ENSMUSG00000118642	*CT010467*	1.048	3.006E-04	-1.100	9.570E-05
ENSMUSG00000090778	*Gm3235*	2.078	3.099E-04	-1.681	9.551E-05

ENSMUSG00000027637	*Rab5if*	-1.534	3.103E-04	1.206	4.439E-04
ENSMUSG00000000693	*Loxl3*	1.208	3.172E-04	-1.068	5.035E-07
ENSMUSG00000108381	*Gm9299*	-1.439	3.246E-04	1.270	1.039E-06
ENSMUSG00000094388	*Gm8783*	-1.769	3.274E-04	1.567	5.618E-04
ENSMUSG00000020424	*Castor1*	-1.317	3.314E-04	1.198	5.821E-05
ENSMUSG00000103677	*Pcdhga4*	2.113	3.538E-04	-1.873	3.102E-08
ENSMUSG00000023064	*Sncg*	1.771	3.661E-04	-1.554	4.372E-04
ENSMUSG00000043889	*Gm8399*	-1.794	3.999E-04	1.392	4.947E-04
ENSMUSG00000097530	*Kansl2-ps*	-1.458	4.160E-04	1.142	2.885E-05
ENSMUSG00000095115	*Itpripl2*	-1.347	4.238E-04	1.064	5.786E-05
ENSMUSG00000042215	*Bag2*	-1.239	4.350E-04	1.095	4.164E-06
ENSMUSG00000102411	*Gm36936*	-2.021	4.386E-04	2.352	1.145E-05

CN, PN, LN, first exposure to CD/HP/HL and re-exposure to normal diet.

Supplementary Table S14. Differentially expressed genes in the testis specific to the high-lard group of female-offspring after re-exposure to HFD in their adult life.

Gene ID	Gene name	LH *vs.* CH		LH *vs.* PH	
		$\mathbf{Log_2}$ **FC**	***P*-value**	$\mathbf{Log_2}$ **FC**	***P*-value**
ENSMUSG00000092511	*Gm20547*	13.795	1.853E-09	13.795	7.320E-10
ENSMUSG00000113120	*E330035G20Rik*	4.916	3.186E-08	4.083	6.511E-05
ENSMUSG00000026840	*Lamc3*	1.751	2.094E-07	1.564	4.318E-05
ENSMUSG00000019970	*Sgk1*	1.463	2.435E-07	1.595	1.113E-05
ENSMUSG00000054074	*Skida1*	-5.286	5.198E-26	-5.047	4.654E-20
ENSMUSG00000030762	*Aqp8*	-2.928	6.641E-12	-2.762	1.106E-05
ENSMUSG00000029272	*Sult1e1*	-16.286	3.739E-11	-20.361	8.061E-14
ENSMUSG00000025329	*Padi1*	-2.307	7.000E-10	-2.254	5.771E-07
ENSMUSG00000019890	*Nts*	-4.103	1.094E-09	-7.691	3.029E-05
ENSMUSG00000112910	*Gm29685*	-11.254	1.874E-08	-10.923	2.957E-08
ENSMUSG00000069355	*Gm5152*	-13.318	1.996E-08	-12.682	7.601E-07
ENSMUSG00000096330	*Gm21976*	-5.018	3.248E-07	-4.828	2.345E-05
ENSMUSG00000032487	*Ptgs2*	-6.427	3.657E-07	-8.158	1.089E-05
ENSMUSG00000023013	*Aqp2*	-3.443	4.361E-07	-3.276	2.103E-05
ENSMUSG00000094584	*Ms4a18*	-11.464	4.796E-06	-11.774	6.684E-06
ENSMUSG00000044626	*Liph*	-2.808	5.276E-05	-4.085	5.510E-06
ENSMUSG00000021403	*Serpinb9b*	-2.773	5.574E-05	-3.589	5.025E-05
ENSMUSG00000114294	*Gm47577*	-10.089	1.141E-04	-11.420	1.127E-08

HFD, high-fat diet. CH, PH, LH, first exposure to CD/HP/HL and re-exposure to high-fat diet.

Supplementary Table S15. Results of Gene Ontology (GO) biological-process enrichment analysis for male PN groups (Figure 5f).

ID	Description	GeneRatio	BgRatio	pvalue	p.adjust	geneID	Count	class
GO:0052695	cellular glucuronidation	1/3	12/28814	0.001248916	0.013344544	Ugt1a5	1	BP
GO:0006063	uronic acid metabolic process	1/3	13/28814	0.001352945	0.013344544	Ugt1a5	1	BP
GO:0019585	glucuronate metabolic process	1/3	13/28814	0.001352945	0.013344544	Ugt1a5	1	BP
GO:0042359	vitamin D metabolic process	1/3	16/28814	0.00166499	0.013344544	Ugt1a5	1	BP
GO:0035988	chondrocyte proliferation	1/3	25/28814	0.002600734	0.014043963	Ltf	1	BP
GO:0006766	vitamin metabolic process	1/3	84/28814	0.008720579	0.020717426	Ugt1a5	1	BP
GO:0001649	osteoblast differentiation	1/3	222/28814	0.022936929	0.031758825	Ltf	1	BP
GO:0009410	response to xenobiotic stimulus	1/3	325/28814	0.033458637	0.040150365	Ugt1a5	1	BP
GO:0008202	steroid metabolic process	1/3	338/28814	0.034781226	0.040570113	Ugt1a5	1	BP
GO:0042581	specific granule	1/4	15/28739	0.00208623	0.010431151	Ltf	1	CC
GO:0055037	recycling endosome	1/4	159/28739	0.021948364	0.05487091	Ltf	1	CC
GO:0005769	early endosome	1/4	327/28739	0.044744447	0.066082276	Ltf	1	CC
GO:0030141	secretory granule	1/4	432/28739	0.05878815	0.066082276	Ltf	1	CC
GO:0045177	apical part of cell	1/4	487/28739	0.066082276	0.066082276	Adam7	1	CC
GO:0004866	endopeptidase inhibitor activity	2/6	216/28275	0.000853913	0.009030079	Ltf/Wfdc15b	2	MF
GO:0030414	peptidase inhibitor activity	2/6	224/28275	0.000917795	0.009030079	Ltf/Wfdc15b	2	MF
GO:0015020	glucuronosyltransferase activity	1/6	34/28275	0.007193834	0.025178418	Ugt1a5	1	MF
GO:0043539	protein serine/threonine kinase activator activity	1/6	58/28275	0.012245826	0.038098124	Ltf	1	MF
GO:0004869	cysteine-type endopeptidase inhibitor activity	1/6	78/28275	0.016439437	0.046030423	Ltf	1	MF
GO:0008194	UDP-glycosyltransferase activity	1/6	149/28275	0.031207134	0.054501003	Ugt1a5	1	MF

Note: GO-BP enrichment analysis of DEGs. GeneRatio represents the proportion of input DEGs assigned to each GO term; BgRatio represents the proportion of genome-wide background genes annotated to each GO term. *p*.adjust, Benjamini-Hochberg adjusted *P*-value.

Supplementary Table S16. Results of Kyoto Encyclopedia of Genes and Genomes (KEGG) pathway enrichment analysis for male PN groups (Figure 5f).

ID	Description	GeneRatio	BgRatio	pvalue	p.adjust	geneID	Count
mmu00053	Ascorbate and aldarate metabolism	1/1	31/9000	0.003444444	0.013333333	Ugt1a5	1
mmu00040	Pentose and glucuronate interconversions	1/1	36/9000	0.004	0.013333333	Ugt1a5	1
mmu00860	Porphyrin metabolism	1/1	43/9000	0.004777778	0.013333333	Ugt1a5	1
mmu00982	Drug metabolism - cytochrome P450	1/1	71/9000	0.007888889	0.013333333	Ugt1a5	1
mmu00980	Metabolism of xenobiotics by cytochrome P450	1/1	73/9000	0.008111111	0.013333333	Ugt1a5	1
mmu00140	Steroid hormone biosynthesis	1/1	92/9000	0.010222222	0.013333333	Ugt1a5	1
mmu00983	Drug metabolism - other enzymes	1/1	92/9000	0.010222222	0.013333333	Ugt1a5	1

Note: KEGG enrichment analysis of DEGs. GeneRatio represents the fraction of input DEGs assigned to each pathway; BgRatio represents the fraction of background genes annotated to each pathway. *p*.adjust: Benjamini-Hochberg adjusted *P*-value. geneID: enriched DEG list; Count: number of enriched DEGs per pathway.

Supplementary Table S17. Results of Gene Ontology (GO) biological-process enrichment analysis for male LH groups (Figure 5g).

ID	Description	GeneRatio	BgRatio	pvalue	p.adjust	geneID	Count	class
GO:0043455	regulation of secondary metabolic process	1/1	15/28814	0.00052058	0.003163145	Slc24a5	1	BP
GO:0048021	regulation of melanin biosynthetic process	1/1	15/28814	0.00052058	0.003163145	Slc24a5	1	BP
GO:0006582	melanin metabolic process	1/1	26/28814	0.000902339	0.003163145	Slc24a5	1	BP
GO:0018958	phenol-containing compound metabolic process	1/1	126/28814	0.004372874	0.006012702	Slc24a5	1	BP
GO:1901617	organic hydroxy compound biosynthetic process	1/1	234/28814	0.008121052	0.009965916	Slc24a5	1	BP
GO:0006813	potassium ion transport	1/1	243/28814	0.0084334	0.009965916	Slc24a5	1	BP
GO:0006814	sodium ion transport	1/1	248/28814	0.008606927	0.009965916	Slc24a5	1	BP
GO:0070588	calcium ion transmembrane transport	1/1	300/28814	0.010411605	0.011452766	Slc24a5	1	BP
GO:0006874	cellular calcium ion homeostasis	1/1	500/28814	0.017352676	0.017352676	Slc24a5	1	BP
GO:0005802	trans-Golgi network	1/1	246/28739	0.008559797	0.010682348	Slc24a5	1	CC
GO:0098791	Golgi apparatus subcompartment	1/1	307/28739	0.010682348	0.010682348	Slc24a5	1	CC
GO:0015368	calcium:cation antiporter activity	1/1	10/28275	0.000353669	0.004871795	Slc24a5	1	MF
GO:0015491	cation:cation antiporter activity	1/1	24/28275	0.000848806	0.004871795	Slc24a5	1	MF
GO:0015298	solute:cation antiporter activity	1/1	29/28275	0.001025641	0.004871795	Slc24a5	1	MF
GO:0015297	antiporter activity	1/1	83/28275	0.002935455	0.010549956	Slc24a5	1	MF
GO:0005262	calcium channel activity	1/1	116/28275	0.004102564	0.010549956	Slc24a5	1	MF
GO:0015085	calcium ion transmembrane transporter activity	1/1	134/28275	0.004739169	0.010549956	Slc24a5	1	MF
GO:0015293	symporter activity	1/1	143/28275	0.005057471	0.010549956	Slc24a5	1	MF
GO:0015081	sodium ion transmembrane transporter activity	1/1	145/28275	0.005128205	0.010549956	Slc24a5	1	MF
GO:0015079	potassium ion transmembrane transporter activity	1/1	157/28275	0.005552608	0.010549956	Slc24a5	1	MF
GO:0046873	metal ion transmembrane transporter activity	1/1	421/28275	0.014889478	0.017046861	Slc24a5	1	MF

Note: GO-BP enrichment analysis of DEGs. GeneRatio represents the proportion of input DEGs assigned to each GO term; BgRatio represents the proportion of genome-wide background genes annotated to each GO term. *p*.adjust, Benjamini-Hochberg adjusted *P*-value.

Supplementary Table S18. Results of Gene Ontology (GO) biological-process enrichment analysis for female PN groups (Figure 5h).

ID	Description	GeneRatio	BgRatio	pvalue	p.adjust	geneID	Count	class
GO:0045121	membrane raft	6/75	393/28739	0.000572524	0.047285414	Vcl/Hspd1/Nedd4/Atp7a/Cys1/Pde2a	6	CC
GO:0098857	membrane microdomain	6/75	394/28739	0.000580189	0.047285414	Vcl/Hspd1/Nedd4/Atp7a/Cys1/Pde2a	6	CC
GO:0005507	copper ion binding	3/74	68/28275	0.000763222	0.094910193	Loxl3/Sncg/Atp7a	3	MF
GO:0052745	inositol phosphate phosphatase activity	2/74	18/28275	0.001006157	0.094910193	Minpp1/Bpnt1	2	MF
GO:0031625	ubiquitin protein ligase binding	5/74	315/28275	0.001429268	0.094910193	Vcl/Laptm4b/Hspd1/Per3/Bag2	5	MF
GO:0044389	ubiquitin-like protein ligase binding	5/74	332/28275	0.001799245	0.094910193	Vcl/Laptm4b/Hspd1/Per3/Bag2	5	MF
GO:0051087	chaperone binding	3/74	109/28275	0.002961251	0.099298057	Hspd1/Atp7a/Bag2	3	MF
GO:0019001	guanyl nucleotide binding	5/74	382/28275	0.003294248	0.099298057	Arl10/Arf6/Gspt1/Gbp10/Pde2a	5	MF
GO:0032561	guanyl ribonucleotide binding	5/74	382/28275	0.003294248	0.099298057	Arl10/Arf6/Gspt1/Gbp10/Pde2a	5	MF

Note: GO-BP enrichment analysis of DEGs. GeneRatio represents the proportion of input DEGs assigned to each GO term; BgRatio represents the proportion of genome-wide background genes annotated to each GO term. *p*.adjust, Benjamini-Hochberg adjusted *P*-value.

Supplementary Table S19. Results of Gene Ontology (GO) biological-process enrichment analysis for female LH groups (Figure 5i).

ID	Description	GeneRatio	BgRatio	pvalue	p.adjust	geneID	Count	class
GO:0010959	regulation of metal ion transport	3/14	446/28814	0.0011809	0.056945616	Ptgs2/Sgk1/Aqp2	3	BP
GO:0007565	female pregnancy	2/14	170/28814	0.003005652	0.062431044	Sult1e1/Ptgs2	2	BP
GO:0010951	negative regulation of endopeptidase activity	2/14	187/28814	0.003621685	0.062431044	Ptgs2/Serpinb9b	2	BP
GO:0030728	ovulation	1/14	23/28814	0.011119822	0.062431044	Ptgs2	1	BP
GO:0008210	estrogen metabolic process	1/14	24/28814	0.011600678	0.062431044	Sult1e1	1	BP
GO:0006883	cellular sodium ion homeostasis	1/14	26/28814	0.012561738	0.062431044	Sgk1	1	BP
GO:0051346	negative regulation of hydrolase activity	2/14	366/28814	0.01323724	0.062431044	Ptgs2/Serpinb9b	2	BP
GO:0014002	astrocyte development	1/14	35/28814	0.016875782	0.067193482	Lamc3	1	BP
GO:0048708	astrocyte differentiation	1/14	84/28814	0.040057927	0.092968664	Lamc3	1	BP
GO:0016702	oxidoreductase activity, acting on single donors with incorporation of molecular oxygen, incorporation of two atoms of oxygen	1/13	29/28275	0.013254385	0.06557626	Ptgs2	1	MF
GO:0043027	cysteine-type endopeptidase inhibitor activity involved in apoptotic process	1/13	35/28275	0.015976344	0.06557626	Serpinb9b	1	MF
GO:0004712	protein serine/threonine/tyrosine kinase activity	1/13	42/28275	0.019143202	0.06557626	Sgk1	1	MF
GO:0022803	passive transmembrane transporter activity	2/13	482/28275	0.019975862	0.06557626	Aqp8/Aqp2	2	MF
GO:0004601	peroxidase activity	1/13	55/28275	0.024999564	0.066746338	Ptgs2	1	MF
GO:0016209	antioxidant activity	1/13	84/28275	0.037947632	0.080844955	Ptgs2	1	MF
GO:0051213	dioxygenase activity	1/13	92/28275	0.041491476	0.084711763	Ptgs2	1	MF
GO:0005179	hormone activity	1/13	132/28275	0.059030642	0.093051253	Nts	1	MF
GO:0099106	ion channel regulator activity	1/13	136/28275	0.060768165	0.093051253	Sgk1	1	MF
GO:0020037	heme binding	1/13	161/28275	0.071560761	0.099877877	Ptgs2	1	MF

Note: GO-BP enrichment analysis of DEGs. GeneRatio represents the proportion of input DEGs assigned to each GO term; BgRatio represents the proportion of genome-wide background genes annotated to each GO term. *p*.adjust, Benjamini-Hochberg adjusted *P*-value.

Supplementary Table S20. Results of Kyoto Encyclopedia of Genes and Genomes (KEGG) pathway enrichment analysis for female LH groups (Figure 5i).

**ID**	**Description**	**GeneRatio**	**BgRatio**	**pvalue**	**p.adjust**	**geneID**	**Count**
mmu05222	Small cell lung cancer	2/8	93/9000	0.00284052	0.059872308	Ptgs2/Lamc3	2
mmu05146	Amoebiasis	2/8	107/9000	0.003742019	0.059872308	Lamc3/Serpinb9b	2

Note: KEGG enrichment analysis of DEGs. GeneRatio represents the fraction of input DEGs assigned to each pathway; BgRatio represents the fraction of background genes annotated to each pathway. *p*.adjust: Benjamini-Hochberg adjusted *P*-value. geneID: enriched DEG list; Count: number of enriched DEGs per pathway.
